# Shared IgG Infection Signatures vs. Hemorrhage-Restricted IgA Clusters in Human Dengue: A Phenotype of Differential Class-Switch *via TGFβ1*

**DOI:** 10.3389/fimmu.2017.01726

**Published:** 2017-12-04

**Authors:** Chung-Hao Huang, Ya-Hui Chang, Chun-Yu Lin, Wen-Hung Wang, Hui-Chung Kuan, Ya-Ju Hsieh, Yu-Wei Wang, Chung-Hsiang Yang, Jhen-Yan Chiu, Shih-Feng Tsai, Yen-Hsu Chen, Hong-Hsing Liu

**Affiliations:** ^1^Division of Infectious Diseases, Department of Internal Medicine, Kaohsiung Medical University Hospital, Kaohsiung, Taiwan; ^2^School of Medicine, Graduate Institute of Medicine, Sepsis Research Center, Kaohsiung Medical University, Kaohsiung, Taiwan; ^3^Institute of Molecular and Genomic Medicine, National Health Research Institutes, Zhunan, Taiwan; ^4^Pediatrics, En Chu Kong Hospital, Sanxia, Taiwan; ^5^Sinying Hospital, Tainan, Taiwan; ^6^Hsieh Te Kuei Pediatric Clinic, HsinChu, Taiwan; ^7^Department of Biological Science and Technology, College of Biological Science and Technology, National Chiao Tung University, HsinChu, Taiwan

**Keywords:** dengue fever, immune repertoire, hemorrhage, class-switch, *TGFβ*, IgG, IgA

## Abstract

Phenotypic manifestations of infectious diseases are closely related to individual immune responses. Methods to extract information from patients’ own immune reactions would be of great use for both diagnosis and treatment. Dengue fever is one of the diseases that clinical aggravations could occur paradoxically after humoral immunity appears. This property makes dengue fever an excellent disease model to explore. A principal component analyses (PCAs)-based framework derived from a prior vaccination study was developed. The framework was verified by successful demonstrations of known IgG signatures from a Mexico Dengue data set. Afterward the pipeline was tested upon *de novo* IgG and IgA libraries of Dengue patients from southern Taiwan. We discovered four infection signatures within IgG repertoires, two of which were identical to previous reports. However, it was IgA but not IgG that could differentiate hemorrhagic from non-hemorrhagic patients. IgA repertoires were found more diversified among bleeders, from whom seven signature clusters were characterized. The expressions of transforming growth factor beta 1 (*TGFβ1*) and accordingly mediated class-switch activity of IgA were distinct only among the PCA-segregated bleeding group. In sum, intercontinental sharing of IgG signatures in dengue fever was demonstrated *via* a unified working flow. Differential regulation of IgA class-switch with associated diversity expansion plus existences of hemorrhage-restricted clusters were shown. The ability of the framework to find common IgG signatures would implicate applications to infections even from unknown pathogens. The clusters within IgA repertoires could offer perspectives to other IgA-related bleeding disorders such as Henoch-Schönlein purpura or IgA nephropathy. Substantiated grounds for IgA-specific effector function *via TGFβ1*-mediated class-switch would be a new factor to consider for infectious diseases.

## Introduction

Humoral immunity could be both friends and foes in human diseases. For example, IgG and IgE antibodies specific for double-stranded DNA could differentially induce pathogenic inflammation in systemic lupus erythematosus (1). Reactions elicited by past viral infections could have effects beyond the same species of viruses (2). Therefore, an effective and versatile pipeline to distil signals out of humoral immunity could be of great value for both basic research and clinical interpretation of human diseases. Dengue fever is an acute febrile illness caused by four groups of dengue viruses (3). Infected mosquitoes of either *Aedes aegypti* or *Aedes albopictus* are the principal vectors in transmitting the pathogens to 4 millions of people in the tropical and sub-tropical areas every year (4). Clinical courses of dengue fever can be divided into three phases: febrile, critical, and convalescent (4). Most patients recover spontaneously, but a few suffer from hemorrhages, plasma leakage, or even circulatory collapses at the critical stage. These severe forms of dengue fever usually occur after the febrile stage, when viral loads are actually very low (5). The most well-recognized factor that significantly increases the likelihood of these serious consequences is the secondary heterotypic infection. Aberrant adaptive immunity might play roles in these scenarios. For example, cross-reactive antibodies against dengue NS1 protein can also induce apoptosis of endothelial cells (6) or enhance activation of plasminogen (7). In addition, the so-called antibody-dependent enhancement (8, 9) hypothesis has been proposed to explain overactivation of myeloid cells after dengue viral infection. Therefore, dengue fever is an excellent disease model for analyses of humoral immunity or antibody repertoires in an infection- and phenotype-specific context.

Recent innovations of next-generation sequencing (NGS) have made possible clonal examinations of adaptive immune responses in dengue fever. Because nucleotides in the complementarity-determining region 3 of the heavy chain (CDR-H3) on most antibodies are sufficient to determine specificities (10), sequence repertoires of this region can effectively serve as clone proxies of humoral immunity. Recently, Parameswaran et al. described convergent IgG signatures among dengue-recovered patients in Nicaragua (11), and Godoy-Lozano et al. found fewer somatic hypermutations among IgG immune repertoires in Mexico (12). It is not clear, although, if there exist specific repertoire signatures that are linked to any of the severe forms of dengue fever.

In this study, we adapted a selection heuristic of antibody repertoires that was developed to characterize carrier children of chronic hepatitis B and vaccination responses of healthy siblings on the platform of NGS (13). After reproducing the same result of the infection signature in the hepatitis B data set by the new pipeline, we applied the renewed scheme to a public data set of dengue fever from Mexico. The infection signatures as reported previously (11, 12) were successfully identified. We then collected blood samples from Taiwan’s Dengue patients. The heuristic indeed revealed four infection signatures, two of which were identical to prior discoveries in Nicaragua and Mexico (11, 12). We further compared IgG and IgA immune repertoires between patients with or without hemorrhages. It was noticed that alterations of diversity profiles in IgA repertoires were more prominent among patients with bleeding phenotypes. Instead IgG repertoires were unable to tell the differences. The identical cluster-seeking heuristic then spotted seven IgA clusters that were closely linked to the clinical bleedings. Furthermore, we found transforming growth factor beta 1 (*TGFβ1*)-mediated class-switch activity of IgA was differentially regulated in the principal component analysis (PCA)-segregated hemorrhagic group only. In conclusion, our results demonstrated an efficient pipeline to characterize the stochastic IgG signatures of viral infection and revealed the hidden relationships between IgA and hemorrhages in dengue fever. Host variations in *TGFβ1*-mediated switch to a more diversified profile of IgA effectors could be a novel contributing factor to dengue hemorrhages.

## Materials and Methods

### Ethics Approval and Consent to Participate

The protocol in recruiting dengue subjects (Table 1; ND_5_, SD_1-7_, and HD_1-7_) was approved by the Institutional Review Board of Kaohsiung Medical University Hospital. Some of non-dengue controls (Table 1; ND_1-4_) were from a previous study (13), the protocol of which was approved by both the Institutional Review Board of National Health Research Institutes and the Institutional Review Board of En Chu Kong Hospital. Study methods were carried out in accordance with the relevant guidelines. Informed consents were obtained from all attendants.

**Table 1:** Clinical profiles of subjects.

ID	Age	Sex	IgG/IgA^a^ timing	Platelet^b^ (10^3^/μL)	AST^b^ (IU/L)	ALT^b^ (IU/L)	Hemorrhage phenotype
ND_1_	42	F	–	–	40	25	–
ND_2_	35	F	–	–	21	14	–
ND_3_	47	F	–	–	30	17	–
ND_4_	37	F	–	–	16	18	–
ND_5_	34	M	− (+1)	–	–	–	–
SD_1_	62	M	2 (+5)	14 (5)	77 (5)	39 (5)	–
SD_2_	71	M	3 (+2)	4 (3)	375 (9)	400 (9)	–
SD_3_	27	M	3 (+2)	89 (3)	221 (3)	169 (3)	–
SD_4_	46	F	3 (+5)	39 (4)	56 (3)	44 (3)	–
SD_5_	63	M	2 (+2)	31 (5)	72 (2)	51 (2)	–
SD_6_	53	F	1 (+6)	79 (6)	152 (5)	92 (5)	–
SD_7_	20	M	2 (+2)	163 (4)	29 (2)	27 (2)	–
HD_1_	56	F	11 (+3)	174 (4)	197 (4)	297 (4)	Ec
HD_2_	64	M	2 (+4)	39 (6)	46 (3)	31 (3)	Pe
HD_3_	54	M	3 (+3)	131 (6)	74 (8)	70 (8)	GI
HD_4_	29	M	3 (+2)	12 (4)	118 (4)	70 (4)	He; GI
HD_5_	35	M	5 (+2)	57 (3)	131 (5)	58 (5)	Pe; Ep
HD_6_	71	F	2 (+3)	6 (7)	209 (5)	180 (3)	Sc; Pe
HD_7_	81	F	4 (+3)	3 (8)	1,349 (5)	543 (5)	Pe

*ND, not dengue; SD, simple dengue; HD, hemorrhagic dengue; AST, aspartate transaminase; ALT, alanine transaminase; Ec, ecchymosis; Pe, petechiae; GI, gastrointestinal bleeding; He, hemoptysis; Ep, epistaxis; Sc, subconjunctival bleeding*.

*^a^Days after dengue onset for first IgG/IgA repertoire and second IgG/IgA repertoire (in parenthesis, plus days following the first repertoire)*.

*^b^Values for lowest plate count and highest AST/ALT on days after dengue onset (in parenthesis)*.

### Clinical Confirmation and Serotyping of Dengue Infection

Blood samples were collected, handled, and discarded in accord with clinical standards. NS1 protein of dengue viruses was tested with the enzyme-linked immunosorbent assay. The viral RNA was extracted from serum with the QIAamp viral RNA kit (Qiagen) according to the manufacturer’s instructions. After reverse transcription, a TagMan real-time PCR was carried out on an ABI thermocycler with a published protocol (14) for serotyping.

### Preparations of CDR-H3 Libraries

Total RNA from 2.5-ml whole blood in the PAXgene tube was extracted with PAXgene Blood miRNA Kit (QIAGEN). 1.2 µg was reverse transcribed by IgG or IgA constant region-specific primers (Table S4 in Data Sheet S2 in Supplementary Material) with SuperScript III Reverse Transcriptase and RNaseOUT (Life Technologies), going through 65°C 5 min, on ice at least 1 min, 55°C 60 min, and final 70°C 15 min.

Normalized cDNA were multiplex amplified with a set of forward V-primers from known IgG alleles (15) that were Primer 3 (16) optimized by the same parameters and two reversed primers (Table S4 in Data Sheet S2 in Supplementary Material). All forward primers shared similar amplification efficiencies with linear correlations to cDNA concentrations across 5 logs and had low backgrounds as well as specific products in pilot optimizations. The reaction condition was 98°C for 2 min, 15 cycles of 98°C for 80 s + 60°C for 60 s + 65°C for 30 s, 10 cycles of 94°C for 30 s + 65°C for 90 sec, and final 65°C for 10 min with AccuPrime *Taq* DNA Polymerase High Fidelity (Life Technologies).

The product was amplified in a second PCR by the same polymerase with double-indexed P5 and P7 primers (Table S4 in Data Sheet S2 in Supplementary Material) under the condition 94°C for 2 min, 10 cycles of 94°C for 30 s + 60°C for 30 s + 68°C for 40 s, 10 cycles of 94°C for 30 s + 72°C for 90 s, and final 72°C for 5 min. Products were sieved by 2% agarose gel under 30 V × 8 h or 60 V × 4 h, and the target bands around 300 bp were eluted with MinElute Gel Extraction Kit (QIAGEN) before 150 bp paired-end sequencing (NextSeq, Illumina, USA).

Demultiplexed raw sequences were processed as reported before (13). Briefly, sequences were paired with PEAR (17); poor quality reads were removed at this step with default parameters, including a Phred filter score at 33. Reads must have no ambiguous “N” nucleotides as well as matched end sequences on both 5′ and 3′ terminals to the amplifying primers. The expected CDR-H3 region as implicated by matched V-primers had to contain multiplicities of three nucleotides without stop codons. The *N*-end of translated amino acids from position 100 to C104 had to align well to anticipated sequences as suggested by matched V-primers and the C-end had to have the W118 as well as the following GXG signatures, where X denotes any amino acids (18). At last, CDR-H3 without the minimal length of two amino acids was discarded.

For CDR-H3 libraries from the Mexico data set, the raw sequencing files were downloaded and subjected to MIXCR alignment (19). Identified CDR-H3 by MIXCR contained the heading C104, trailing W118, and occasional non-amino acid letters like “*” or “_” in between. We preprocessed these CDR-H3 by trimming C104/W118 and discarding “*” or “_” containing reads before bridging to the downstream analyses.

### Real-Time PCR for *TGFβ1* and IgA Germ-Line Transcripts (GLTs)

cDNA was synthesized with random hexamers primed to the same RNA used in preparations of CDR-H3 libraries. *TGFβ1* was qPCR-assayed (KAPA SYBR FAST Master Mix, Kapa Biosystems) with a published primer pair (20), 5′-CCCAGCATCTGCAAAGCTC-3′ and 5′-GTCAATGTACAGCTGCCGCA-3′, under the condition 95°C for 3 min, 40 cycles of 95°C for 1 s + 60°C for 25 s, and a final dissociation stage on 7900HT Fast Real-Time PCR System (Applied Biosystems). IgA GLT was first PCR amplified from RNase-H cleaned cDNA with primers 5′-CAGCAGCCCTCTTGGCAGGCAGCCAG-3′ and 5′-TTTCGCTCCAGGTCACACTGA-3′ (21) under the condition 94°C for 2 min, 14 cycles of 94°C for 30 s + 60°C for 30 s + 68°C for 30 s, and 68°C for 5 min with AccuPrime Taq DNA Polymerase High Fidelity (Thermo Fisher Scientific). The product was then nested qPCR-assayed (KAPA SYBR FAST Master Mix, Kapa Biosystems) with primers 5′-TTGGCAGGCAGCCAGACG-3′ and 5′-TGGGGCTGGTCGGGGATG-3′ under the condition 95°C for 3 min, 40 cycles of 95°C for 1 s + 60°C for 20 s, and a final dissociation stage with the same qPCR reagent and instrument as used for *TGFβ1*. The nested primer pair was based on Sanger sequences obtained from outer PCR products, and the final nested products were confirmed by Sanger sequencing as well. Cycles of thresholds (Cts) for both transcripts were normalized to Ct of *Actin*. Expressions of *TGFβ1* and GLT were compared with Mann–Whitney *U* and Student’s *t*-tests, respectively. Of note, GLT comparisons excluded patient SD_6_ to control the time spans between first and second samples within 5 days (Table 1). The significance of the *p* value of 0.48 for the hemorrhage-absent central group was found <0.001 by fitting against the *p* value distribution exhaustively built from all *p* values at *n* = 5 from the hemorrhage-prevalent peripheral group.

### Real-Time PCR for IgA1 vs. IgA2 Subclasses

IgA-specific first-strand cDNA from patient HD_3_ as used for preparations of CDR-H3 libraries was assayed with the following primers to quantify total IgA1 vs. IgA2 and cluster-specific IgA1 vs. IgA2. For the former, a common forward primer 5′-ACCAGCCCCAAGGTCTTCC-3′ was paired with 5′-GATGACCACGTTCCCATCTG-3′ and 5′-GACGACCACGTTCCCATCTT-3′ reverse primers to detect total IgA1 and IgA2, respectively. A cluster-specific forward primer 5′-TGCGACGGTCTTCACTACAG-3′ was chosen among the most abundant CDR-H3 sequences of cluster ATVFTTVHY to examine cluster-specific IgA1 and IgA2 with the same reverse primers above. The reactions were performed in triplicates with KAPA SYBR FAST qPCR Master Mix (Kapa Biosystems) on 7900HT Fast Real-Time PCR System (Applied Biosystems) under the condition 95°C for 3 min, 40 cycles of 95°C for 1 s plus 60°C for 10 s, and final addition of a dissociation stage to confirm product specificity.

### Bioinformatic Segregation of IgG Subclasses

3′ extension sequences after the two reverse primers paired with V-primers (Table S4 in Data Sheet S2 in Supplementary Material) were used to classify NGS reads into IgG subclasses. 5′-GCCCTTGGTGGAG-3′ would categorize reads into the composite group of IgG1 + IgG2, while 5′-GCCCTTGGTGGAA-3′ would segregate reads into the other group of IgG3 + IgG4. Relative percentages of cluster-specific reads among both groups were calculated for all dengue samples. The results of two series were subjected to the Mann–Whitney *U* test, which yielded *p* = 0.28.

### Principal Component Analysis

The normalized clone frequencies for each sample were Hellinger transformed (22) before PCA (23) in SciPy (24), where “svd_solver” was set to “full.” Clones ranked beyond 2 SDs in absolute values of loadings were used in clustering as specified in the text and below.

### Rarefaction and CDR-H3 Clustering

Samples with more reads were randomly resampled with Python to match the repertoire with minimal read counts in the indicated data set. Rarefactions were performed 10 times and saved for downstream analyses. Clusters were identified in two steps. Indel-free Hamming distances (25) between clone pairs were calculated into an adjacency matrix in Python, which was used to initiate a graph in igraph (26) discarding edges weighted higher than distance 1. Cluster-associated functions in igraph were applied to discover independent clusters and to calculate associated PageRank scores (27).

### Morisita Index

For repertoire *A* and *B* with clone frequencies *F_A_* and *F_B_*, the Morisita dissimilarity index (28) is a measure to quantify the distance between two sets of clone sequences. This measure is affected mainly by abundant clones; relatively rare clones have little effect, even if there are many of them. The measure (*M*) was calculated as follows:

$$M=1-\frac{2\sum (F_{A}F_{B})}{\sum F_{A}{\;}^{2}+\sum F_{B}{\;}^{2}}$$

### Diversity Profiles by Hill Numbers

In each rarefaction data set, samples with the same hemorrhagic phenotype and immunoglobulin class were pooled. Within each pool, clusters were defined by an adjacency matrix as described above. We then used Hill numbers, i.e., effective number of clusters in this case, to quantify cluster diversity. Diversity profiles in Hill numbers (*D*) (29) were calculated following the formula with frequencies normalized in each pool:

$$D_{q}={\left(\sum_{C}f^{q}\right)}^{1/(1-q)}\{\begin{array}{l} C,\text{cluster}\\ f,\text{cluster frequency}\\ q,\text{Hill number parameter} \end{array}$$

The parameter *q* determines each measure’s sensitivity to normalized frequencies. The measure of *q* = 0 (the total diversity) counts clusters equally without regards to their normalized frequencies. The measure of *q* = 1 [Shannon diversity, the exponential of Shannon entropy (30)] counts clusters in proportional to their normalized frequencies and thus can be interpreted as the number of common clusters in the data. The measure of *q* = 2 discounts all but the dominant clusters and can be interpreted as the number of dominant clusters in the data. The plot of *D_q_* with respect to the parameter *q* is referred to as a “diversity profile” in ecological science. The profile is generally a decreasing function. The slope of the curve reflects the unevenness of cluster-normalized frequencies. The more uneven the distribution of normalized frequencies, the more steeply the curve declines. Graphs with *q* = (0, 5] were generated with igraph (26).

### Construction of Clone Pools for Cluster Build-up

In this study, significantly contributing clones in PCA were selected as building blocks for clusters. In the previous work of chronic hepatitis B (13), carrier and non-carrier clone pools were instead made of 2-occurrence CDR-H3 sequences among the four carriers and the four non-carriers, respectively, regardless of vaccination histories. Clusters were then defined among the indicated clone pools with adjacency matrices before downstream feature selections.

### Cluster Selection for Hepatitis B and Dengue Infections

**Figure 1:**
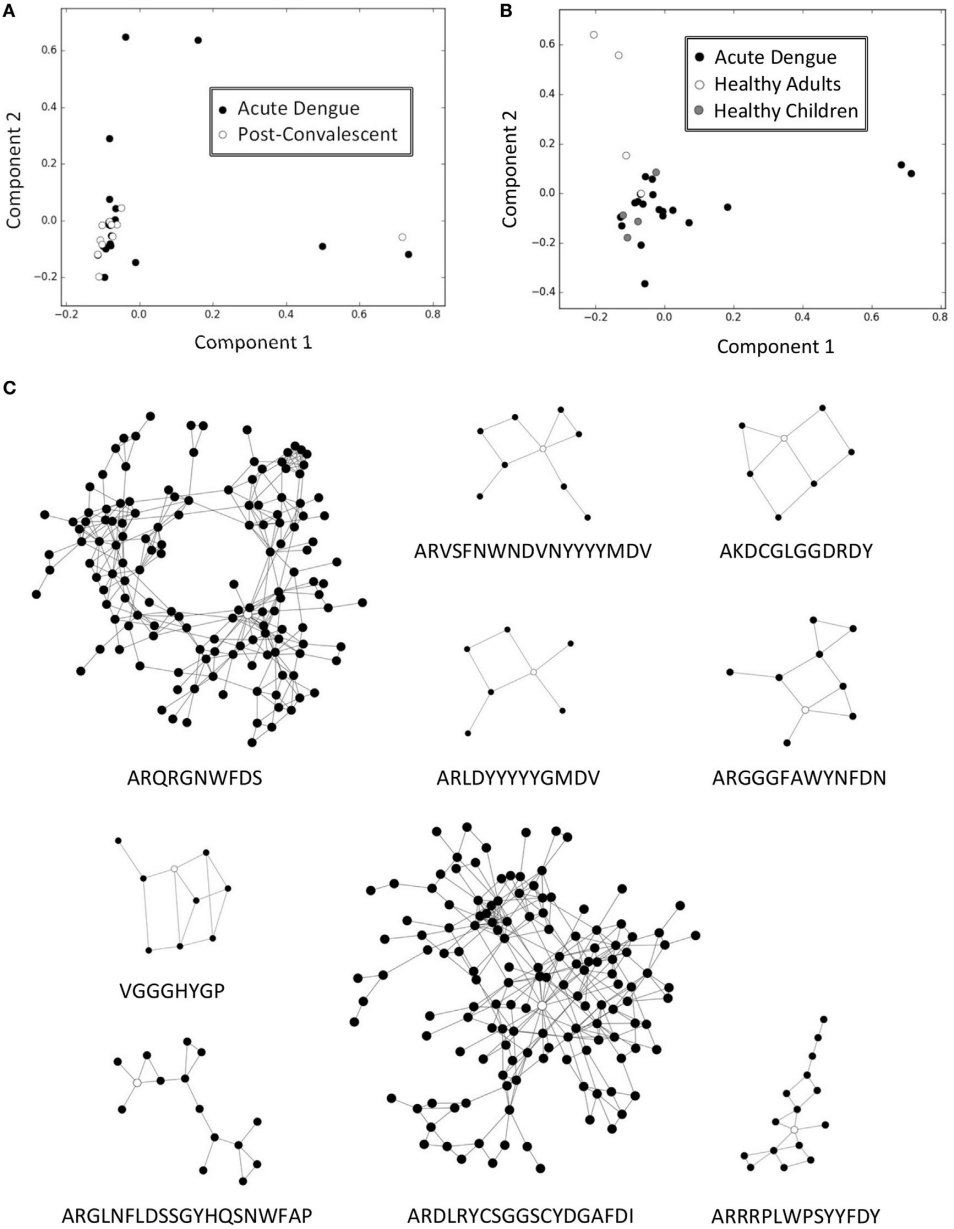
IgG infection signatures of dengue fever in Mexico. **(A)** IgG immune repertoires of acute and post-convalescent dengue fever in Mexico could not be separated on the principal component analysis (PCA) plot. **(B)** IgG immune repertoires of acute dengue in Mexico could be fairly separated from healthy adults in Taiwan but not from children controls. **(C)** Nine IgG clusters were identified as infection signatures from those clones with highest loading values in PCA of mixed Mexico and Taiwan samples. Vertices represented sequence clones of complementarity-determining region 3 of the heavy chain and connected edges denoted indel-free Hamming distance one of amino acids between clones. The sequences with the highest PageRank scores in the indicated clusters were used as the representatives, including ARQRGNWFDS, VGGGHYGP, ARGLNFLDSSGYHQSNWFAP, ARVSFNWNDVNYYYYMDV, AKDCGLGGDRDY, ARDLRYCSGGSCYDGAFDI, ARRRPLWPSYYFDY, ARLDYYYYYGMDV, and ARGGGFAWYNFDN; these nine sequences were marked as hollow vertices in the indicated clusters.

**Figure 2:**
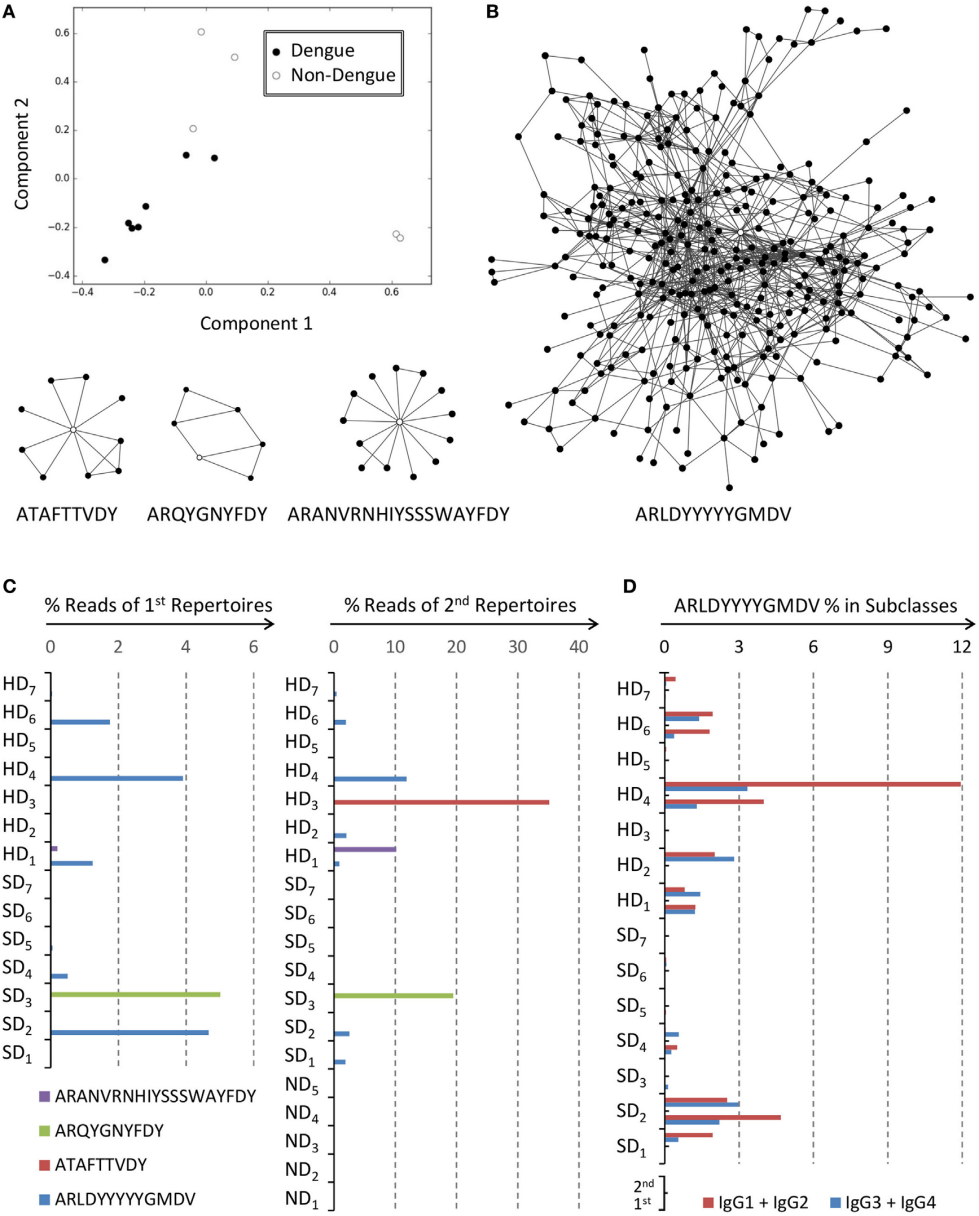
IgG infection signatures of dengue fever in Taiwan. **(A)** Principal component analysis (PCA) of IgG immune repertoires distinguished dengue patients from non-dengue subjects. **(B)** Four IgG clusters were identified as infection signatures from those clones with highest loading values in PCA. Vertices represented sequence clones of complementarity-determining region 3 of the heavy chain (CDR-H3), and connected edges denoted indel-free Hamming distance one of amino acids between clones. The sequences with the highest PageRank scores in the indicated clusters were used as the representatives, including ATAFTTVDY, ARQYGNYFDY, ARANVRNHIYSSSWAYFDY, and ARLDYYYYYGMDV; these four sequences were marked as hollow vertices in the indicated clusters. **(C)** The sequence reads of cluster ARLDYYYYYGMDV were prevalent among most dengue patients. Instead sequence reads of cluster ATAFTTVDY, ARQYGNYFDY, and ARANVRNHIYSSSWAYFDY were limited to specific individuals. The latter two clusters were detectable in the first IgG repertoires already for the indicated patients. **(D)** IgG reads from both time points were bioinformatically segregated into IgG1 + IgG2 or IgG3 + IgG4 composite groups. Percentages of reads belonging to cluster ARLDYYYYYGMDV were calculated for all dengue patients. There were no significant preferences to either group by Mann–Whitney *U* test (*p* = 0.28).

Linear support vector classification (SVC) and logistic regression (LR) from Scikit-learn (31) were used to select clusters with “l1” penalties. Hellinger transformed frequencies served as independent variables for classification models. The top 1 or 0.5% of clusters with most abundant members were candidate clusters subjected to selections. In choosing clusters marking Dengue or hepatitis B infections (Figures 1C and 2B; Figure S1B in Data Sheet S1 in Supplementary Material), repertoires from confirmed patients were labeled positive. For hemorrhage-associated clusters, repertoires from bleeding patients were marked positive. Regulatory parameters were optimized by leave-one-out cross validations. Clusters were selected if both SVC and LR models gave positive supports. In rarefied experiments, clusters that manifested only in one data set were discarded.

### Availability of Data

The data set of Mexico Dengue samples is available at NCBI-SRA repository, BioProject ID PRJNA302665. The data sets of Taiwan samples for healthy controls and dengue samples are available at European Nucleotide Archive, PRJEB9332 and PRJEB13768, respectively.

## Results

### Variation-Contributing Clones for Discoveries of Antibody Repertoire Signatures

A previous study in characterizing antibody repertoires among carrier children of chronic hepatitis B found that PCA (23) could readily separate carrier from non-carrier repertoires in a non-supervised manner (13). We hypothesized that those CDR-H3 sequence clones (briefed as clones in the following text) with higher absolute loading values in PCA would be sufficient to spot the infection signature (13) (Figure S1A in Data Sheet S1 in Supplementary Material). We identified 20,429 and 41,106 clones from carriers and non-carrier repertoires, respectively, that had absolute loading values beyond 2 SDs in the PCA. These clones were each categorized by one amino acid difference criterion, i.e., Hamming distance 1 without indels (25), into 17,392 and 32,083 clusters, respectively. The top 0.5% of clusters with most abundant members in either category were subjected to feature selections by both SVC and LR. Among a total of 246 clusters, only 1 (Figure S1B in Data Sheet S1 in Supplementary Material) gained very strong supports from both models (Table S1 in Data Sheet S2 in Supplementary Material), and it happened to be the previously published infection signature (13) comprising 28 unique clones (Figure S1C in Data Sheet S1 in Supplementary Material). We expected that the same selection scheme could be generalized to other diseases as well. The idea was tested with a public data set of dengue fever from Mexico (12). We found that repertoires from acute dengue and postconvalescent samples overlapped to a large extent on the PCA plot (Figure 1A). To improve the contrast, we mixed IgG repertoires from 4 healthy adults and 4 healthy children in the vaccination study (13) and 19 acute dengue samples from the Mexico data set. We further conducted 10 runs of rarefactions to match the lower sequencing depths in the dengue data set. Effectively the acute dengue repertoires separated fairly from those of adults but not from children (Figure S2 in Data Sheet S1 in Supplementary Material). A typical PCA plot from one of the rarefied data set is shown (Figure 1B). Candidates were set to the top 1% of member-rich clusters as constructed from those clones contributing variations most on PCA plots. Intersected results from both SVC and LR models (Table S2 in Data Sheet S2 in Supplementary Material) yielded nine signature clusters (Figure 1C), which appeared at least twice among 10 rarefactions (Table S3 in Data Sheet S2 in Supplementary Material). Each cluster was designated with the clone sequence that had the highest PageRank score (27). There were 143, 9, 15, 9, 7, 140, 16, 7, and 9 members for cluster ARQRGNWFDS, VGGGHYGP, ARGLNFLDSSGYHQSNWFAP, ARVSFNWNDVNYYYYMDV, AKDCGLGGDRDY, ARDLRYCSGGSCYDGAFDI, ARRRPLWPSYYFDY, ARLDYYYYYGMDV, and ARGGGFAWYNFDN, respectively (Table S3 in Data Sheet S2 in Supplementary Material). The most prevalent cluster, ARQRGNWFDS, occurred in all rarefactions (Table S3 in Data Sheet S2 in Supplementary Material) and was the same as reported by the original dataset contributors (12). One of the clusters, ARLDYYYYYGMDV, was the convergent dengue signature reported by Parameswaran et al. (11). On the basis of these establishments, we set out to investigate antibody repertoires of dengue fever in Taiwan.

### NGS Study on Dengue Fever in Taiwan

15 feverish patients from a single medical center in southern Taiwan were recruited to the study (Table 1; ND_5_, SD_1-7_, and HD_1-7_). Of the 15 patients, 14 had dengue NS1 protein detected in blood, but the other did not. Half of the dengue-confirmed cases had clinical evidences of hemorrhages plus other signs like thrombocytopenia or elevated hepatic transaminases (Table 1; HD_1-7_), meeting at least the criterion of dengue fever with warning signs (4) in terms of severity. However, none of the recruited subjects suffered from plasma leakage or circulatory collapses. All of the 15 patients contributed blood samples at two different time points (Table 1; referenced from the disease onset). There were no significant differences of age, sex, first sampling day, second sampling day, lowest platelet count, and highest AST or ALT values between both groups (ANOVA, minimal *p* = 0.13).

We prepared CDR-H3-based immune repertoires with optimized PCR primers (Table S4 in Data Sheet S2 in Supplementary Material) for parallel sequencing. On the basis of total RNA without enrichments, we got average reads of 792,819 ± 475,770 (SD) for IgG libraries and 847,278 ± 442,526 (SD) for IgA libraries; unique sequence clones amounted to 45,498 ± 20,894 (SD) for IgG repertoires and 52,033 ± 22,358 (SD) for IgA repertoires, respectively (Table S5 in Data Sheet S2 in Supplementary Material). All comparisons between hemorrhagic and non-hemorrhagic patients in terms of counts of IgG reads, IgG clones, IgA reads, and IgA clones at both time points yielded insignificant results except the counts of IgG clones at the second time point (Student’s *t*-test, *p* = 0.02; minimal *p* = 0.29 for the other comparisons).

### Network Signatures of IgG Repertoires

To identify infection signatures of dengue fever in Taiwan, the four IgG adult control repertoires as used above with the Mexico data set were again combined with eight dengue IgG repertoires in a strict chronological order of case recruitment, without exclusions, for PCAs. The repertoires at the second time point for each dengue patient were adopted because longer infection exposures presumably would better mark antibody repertoires. We found that dengue repertoires could be fairly separated from healthy controls (Figure 2A). The two components explained 10.1 and 9.4% variances, respectively. A set of 13,548 unique clones whose absolute loading values passed beyond 2 SDs in PCA was grouped into 10,853 clusters with the Hamming distance 1 criterion without indels. The top 1% of clusters with most abundant members were subjected to feature selections by both SVC and LR. Clusters were selected if both SVC and LR models gave positive supports. We found four clusters satisfied the above filtering conditions (Table S6 in Data Sheet S2 in Supplementary Material). Each cluster was designated with the clone sequence that had the highest PageRank score (27). There were 321, 10, 6, and 14 members for cluster ARLDYYYYYGMDV, ATAFTTVDY, ARQYGNYFDY, and ARANVRNHIYSSSWAYFDY, respectively (Table S7 in Data Sheet S2 in Supplementary Material). Cluster ARLDYYYYYGMDV and ARQYGNYFDY were reported before (11, 12), but the others were not. Except cluster ARLDYYYYYGMDV, topographies of all other three clusters were relatively simple (Figure 2B). We calculated the percentages of NGS reads for each cluster in the data set (Figure 2C). Although the four clusters were selected from a half of dengue patients who were recruited earlier, the other half of patients also had significant percentages of readings for cluster ARLDYYYYYGMDV (Figure 2C). Many of the patients had that cluster in their IgG repertoires at the first time point already (Figure 2C). Cluster ATAFTTVDY, ARQYGNYFDY, and ARANVRNHIYSSSWAYFDY were not shared among subjects, but the latter two had been detectable in the first IgG repertoires for indicated patients (Figure 2C). Non-dengue controls had no discernible presence of any of these four clusters in their IgG repertoires (Figure 2C). We further segregated members of cluster ARLDYYYYYGMDV into two composite groups of either IgG1 + IgG2 or IgG3 + IgG4. The same categorization was also performed on all IgG reads at both time points. Relative percentages for cluster ARLDYYYYYGMDV among both groups were illustrated (Figure 2D). There were no subclass preferences with p-value at 0.28 by Mann–Whiney *U* test.

### More Diversified IgA Repertoires in Patients with Hemorrhages

**Figure 3:**
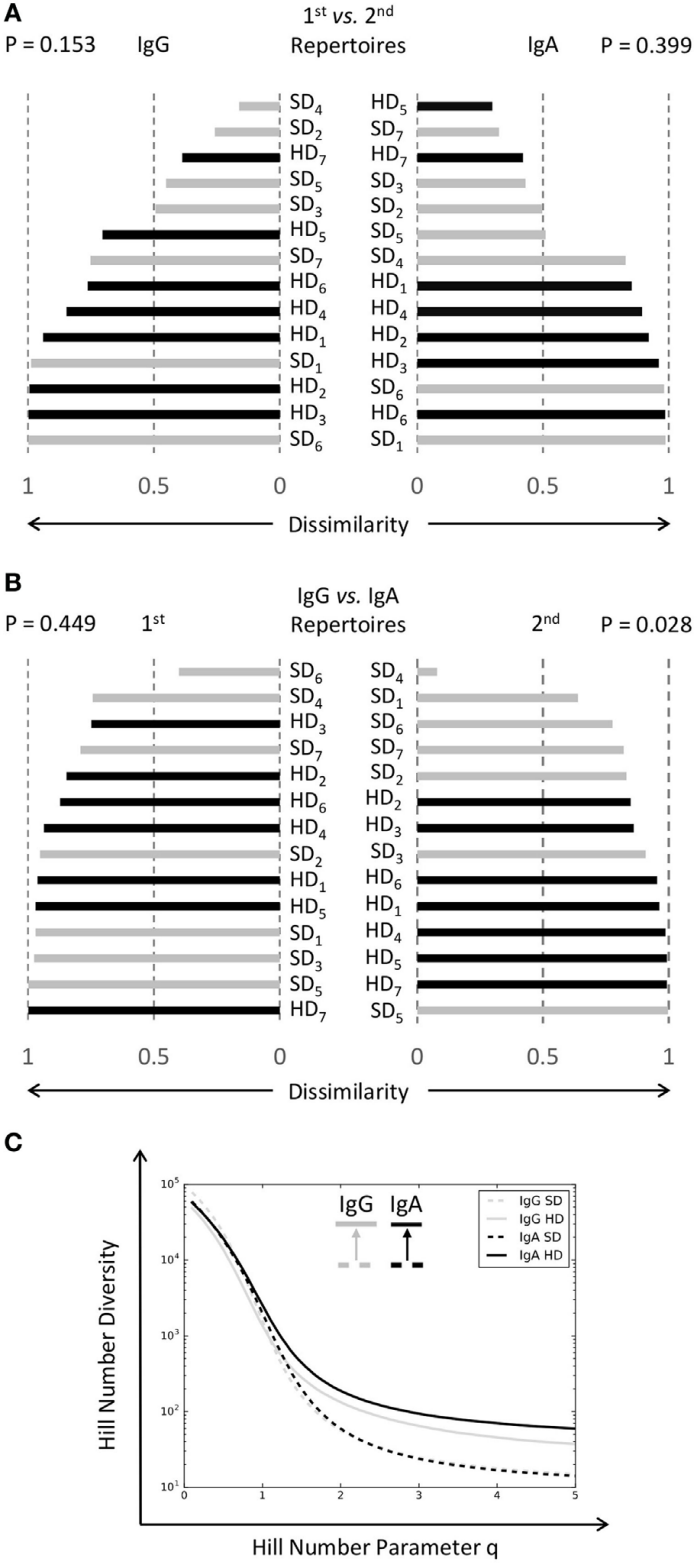
Distinct IgA immune repertoires in hemorrhagic dengue patients. **(A)** Morisita indices between immune repertoires of the same class at both time points were calculated. Black bars denoted results from bleeding patients (HD_1-7_), while gray bars represented those from non-bleeding patients (SD_1-7_). There were no significant differences for either IgG (*p* = 0.153, *U* test) or IgA (*p* = 0.399, *U* test). **(B)** Morisita indices between IgA and IgG immune repertoires were calculated at both time points. Among hemorrhagic patients (HD_1-7_), the relative dissimilarities between IgA and IgG were higher than those from non-hemorrhagic patients (SD_1-7_) at the second time point (*p* = 0.449 for first and *p* = 0.028 for second, *U* test). The difference was visually discernible by more black bars at the lower part of the right half-figure. **(C)** Diversity profiles of Hill numbers based on IgG and IgA immune repertoires were plotted. For both classes of immunoglobulins, hemorrhagic patients had higher diversities than non-hemorrhagic patients, as depicted by the upward trends of diversity curves. The difference was more prominent for IgA immune repertoires.

IgA levels in body fluids are known to fluctuate along the course of dengue fever (32, 33). Because cross-reactive IgG antibodies have been reported to cause undesired consequences (6, 7), the pathogenic roles that might be attributable to IgA immunoglobulins are worth further investigations. First, we used Morisita index (28) to compare dissimilarities between the same isotypes for each dengue patient at both time points (Figure 3A). We did not see significant differences for either type of immunoglobulins, although hemorrhagic patients seemed to have higher dissimilarities for IgA (Figure 3A). We then compared dissimilarities between IgG and IgA antibodies (Figure 3B). The results showed that IgG and IgA were more dissimilar for those patients with hemorrhages (Figure 3B). The average Morisita indices for bleeders and non-bleeders were 0.92 ± 0.02 (SE) and 0.78 ± 0.07 (SE), respectively. Mann–Whitney *U* test exhibited a significant difference (*p* = 0.028). A numerical assay to absolutely evaluate the status shifts of IgA immune repertoires can provide more insights than relative comparisons between IgG and IgA alone. Diversity profiles in terms of Hill numbers that are widely used in numerical ecology (29) could be useful in this context (13, 34).

10 runs of random rarefactions were conducted to normalize NGS reads among different samples. In each rarefied data set, samples were pooled together by both the clinical phenotypes of hemorrhages vs. non-hemorrhages and immunoglobulin classes of IgG vs. IgA. Diversity profiles in Hill numbers were plotted for each data set (Figure S3 in Data Sheet S1 in Supplementary Material), where clusters as defined by the indel-free Hamming distance 1 criterion were compared to ecological species in the calculation (29). All of the plots were nearly identical (Figure S3 in Data Sheet S1 in Supplementary Material), and the one from rarefied data set 1 was illustrated (Figure 3C). Upward shifts of the curves from hemorrhagic patients were noted for both IgG and IgA, but the latter apparently had a stronger momentum (Figure 3C). The trend was especially prominent for more abundant clusters as revealed by enlarging gaps along the increasing parameter of Hill numbers (Figure 3C). Therefore, the bleeding phenotypes in dengue patients were associated with a diversity expansion of immunoglobulins, of which IgA seemed to be more dominant.

### Differential IgA Repertoires in Patients with Hemorrhages

**Figure 4:**
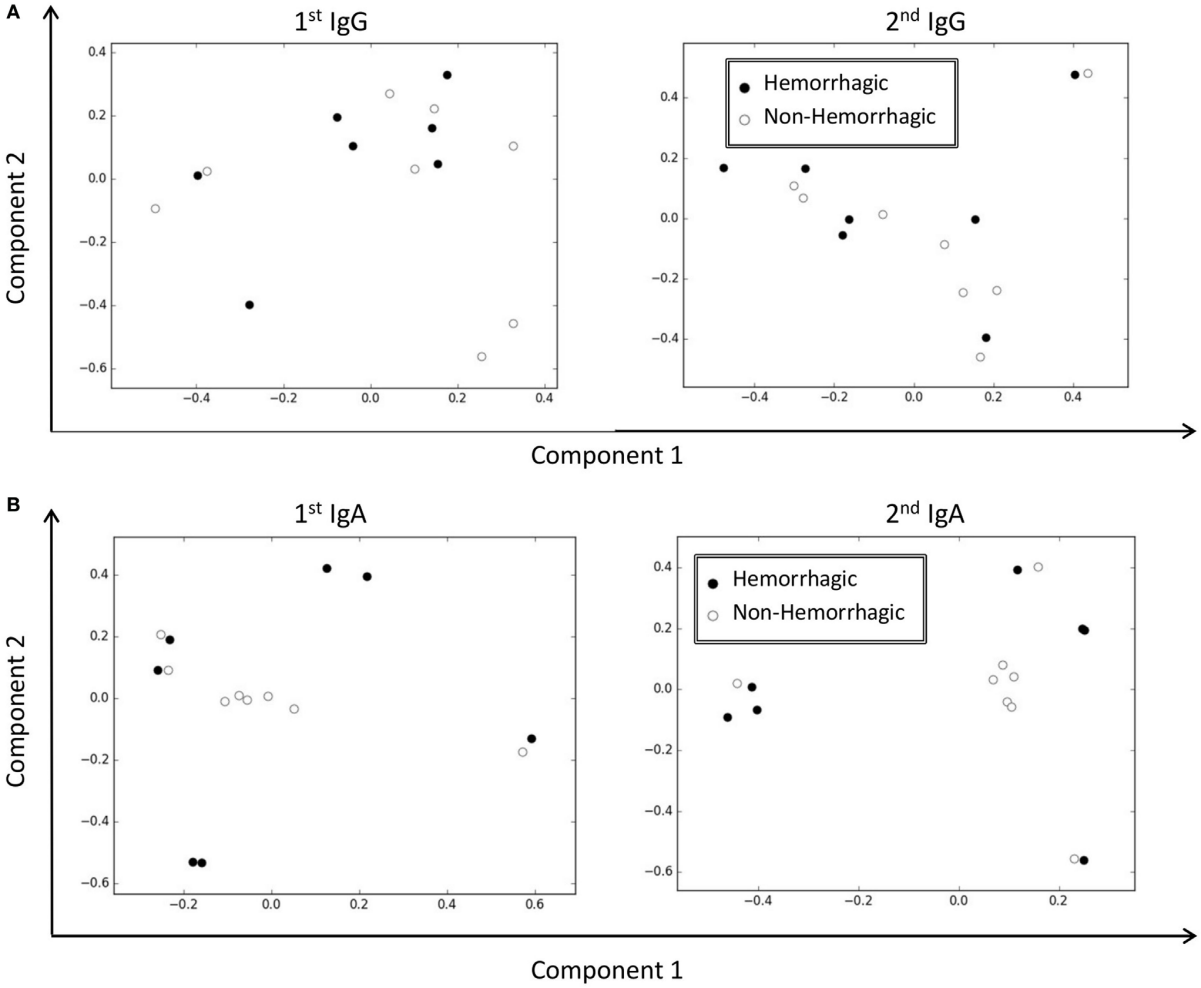
Principal component analysis (PCA) of IgA immune repertoires distinguished dengue fever with hemorrhages. **(A)** PCA analyses of IgG immune repertoires at either time point failed to separate hemorrhagic patients from non-hemorrhagic patients. **(B)** Both IgA immune repertoires could clearly group patients in accord with phenotypes. Most non-hemorrhagic patients took central positions in the PCA plots, but all hemorrhagic patients were located in the periphery.

With differential diversity shifts of IgG and IgA in hemorrhagic patients (Figure 3C), we hypothesized that these two classes of immunoglobulins might relate differentially to the bleeding phenotypes. We used PCA to summarize IgG and IgA variations among all 15 patients with samples at both time points (Table 1; ND_5_, SD_1-7_, and HD_1-7_). We found IgG repertoires at either time point were unable to classify patients in accord with their hemorrhagic phenotypes (Figure 4A). IgA repertoires instead were distinctly clustered into several groups; most non-hemorrhagic patients were clearly aggregated to the central area on PCA plots (Figure 4B), including the NS1-negative patient ND_5_. Clustering in PCA coordinates were significant at *p* = 0.001 by the PERMANOVA test (35).

To stress the clustering power, we did IgA PCA with the 10 rarefied data sets as prepared above (Figure S4 in Data Sheet S1 in Supplementary Material). Although the repertoires from the first time point became suboptimal in resolving patient groups, the repertoires from the second time point were still as robust as those from the full data set (Figure 4B). Therefore, the clones with high loading values in PCAs as constructed from the second IGA repertoires could be closely associated with the hemorrhagic phenotypes.

### Network Signatures of IgA Repertoires in Patients with Hemorrhages

**Figure 5:**
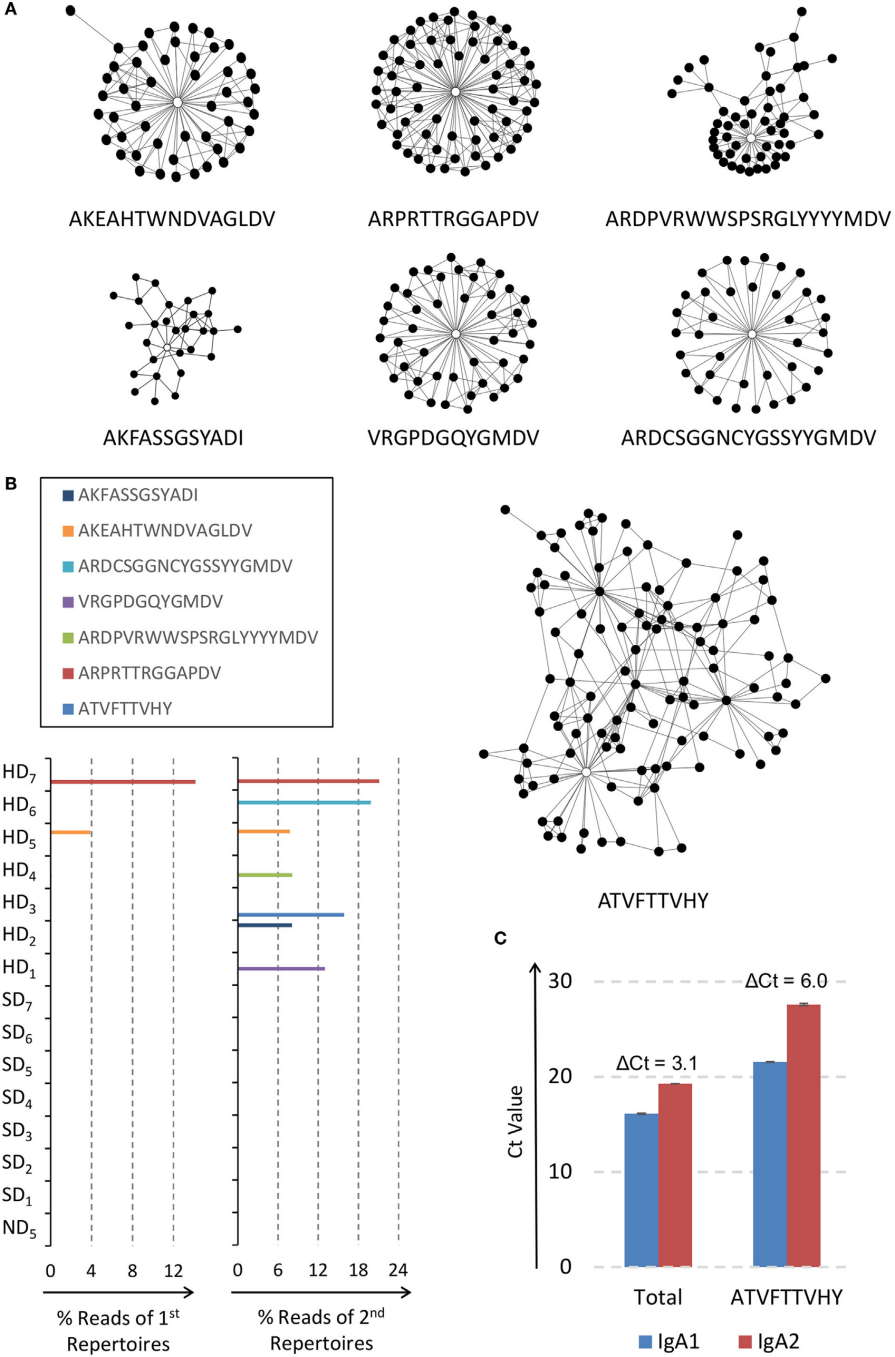
Network signatures of IgA immune repertoires among hemorrhagic dengue patients. **(A)** Seven IgA clusters were closely associated with hemorrhages. Vertices represented sequence clones of complementarity-determining region 3 of the heavy chain and connected edges denoted indel-free Hamming distance one of amino acids between clones. The sequences with the highest PageRank scores in the indicated clusters were used as the representatives, including ATVFTTVHY, ARPRTTRGGAPDV, ARDPVRWWSPSRGLYYYYMDV, VRGPDGQYGMDV, ARDCSGGNCYGSSYYGMDV, AKEAHTWNDVAGLDV, and AKFASSGSYADI; these seven sequences were marked as hollow vertices in the indicated clusters. **(B)** Sequence reads of all seven clusters were only present among hemorrhagic dengue patients. None of the clusters were shared between each other. Clusters AKEAHTWNDVAGLDV and ARPRTTRGGAPDV were already detectable in the first IgA immune repertoires of indicated patients. **(C)** Results of real-time PCR in triplicates to quantify total IgA1 vs. IgA2 and cluster ATVFTTVHY-specific IgA1 vs. IgA2 upon the second sample of patient HD_3_. Both had IgA1 predominance, although the cycle of threshold (Ct) value gap was smaller for total IgA (ΔCt = 3.1) than cluster-specific IgA (ΔCt = 6.0).

We took a similar strategy used in identifying infection signatures of IgG repertoires (Figures 1 and 2) to analyze those clones with highest absolute loading values in PCAs of the second IgA repertoires. For each data set, these clones were clustered first with the indel-free Hamming distance 1 criterion (29). The top 0.5% clusters with most abundant clone members were subjected to both SVR and LR feature selections. We found seven clusters that gained strong supports from both SVR and LR models in all 10 rarefactions (Table S8 in Data Sheet S2 in Supplementary Material). Each cluster was designated with the clone sequence carrying the highest PageRank score (27) among pooled CDR-H3 sequences of the indicated cluster from all 10 data sets (Figure 5A). The member counts for cluster ATVFTTVHY, ARPRTTRGGAPDV, ARDPVRWWSPSRGLYYYYMDV, VRGPDGQYGMDV, ARDCSGGNCYGSSYYGMDV, AKEAHTWNDVAGLDV, and AKFASSGSYADI were 106, 66, 53, 47, 38, and 50, respectively (Table S9 in Data Sheet S2 in Supplementary Material). Only among hemorrhagic patients did clusters have high read percentages in the second IgA repertoires (Figure 5B). Cluster ARPRTTRGGAPDV and AKEAHTWNDVAGLDV were already detectable in the first IgA repertoires for indicated patients. There was no sharing of clusters among hemorrhagic patients. Of note, IgA cluster ATVFTTVHY (Figure 5A) and IgG cluster ATAFTTVDY (Figure 2B) were essentially related (Tables S7 and S9 in Data Sheet S2 in Supplementary Material), but the topology in the latter (Figure 2B) was much simpler. We suspected that differential class-switch activity of IgA might be the underlying driving force for the appearance of the more complex IgA variant along with more diversified IgA profile (Figure 3C) among the bleeding patients.

### Differential Regulation of *TGFβ1*-mediated IgA Class-Switch in PCA-Segregated Group with Hemorrhages

**Figure 6:**
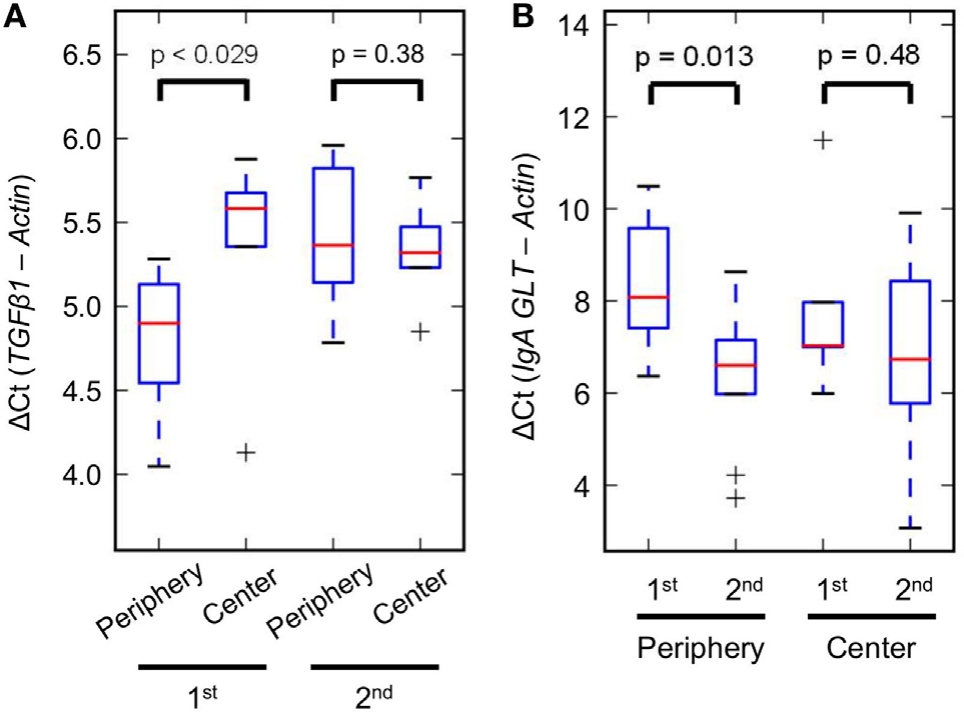
Differential regulation of transforming growth factor beta 1 (*TGFβ1*)-mediated IgA class-switch activity. **(A)** *TGFβ1* expressions of the hemorrhage-prevalent peripheral group on principal component analysis were higher than those of the hemorrhage-absent central group at disease onset (first samples), but became comparable a few days later (second samples). **(B)** Germ-line transcript (GLT) levels, which represent class-switch activity of IgA, were increased shortly after disease onset for the peripheral group, but instead kept constant for the central group.

We first used real-time PCR to quantify IgA subclasses. Cluster ATVFTTVHY-specific IgA1 vs. IgA2 was compared with total IgA1 vs. IgA2 on the second sample of patient HD_3_ (Figure 5C). Results showed that IgA1 was the more dominant subclass. The difference of Ct for total IgA was 3.1, but the Ct gap for cluster ATVFTTVHY got higher to 6.0 (Figure 5C). We then set out to assay global switch activity of IgA as induced by *TGFβ* (36). Dengue patients with hemorrhages are known to have higher expressions of *TGFβ* (37). We used qPCR to compare blood *TGFβ1* levels between the hemorrhage-prevalent peripheral group on the IgA PCA plot (Figure 4B and Table 1; SD_1,6,7_, and HD_1-7_) and the hemorrhage-absent group at the center (Figure 4B and Table 1; SD_2-5_ and ND_5_). Indeed shortly after dengue onset the peripheral group had much higher *TGFβ1* levels than the central group (Figure 6A; *p*< 0.029, *U* test). However, the difference was no longer discernible in a few days (Figure 6A; *p* = 0.38, *U* test). Because *TGFβ1* is an IgA-specific class-switch factor (36) through inductions of IgA GLTs (38), we tested the class-switch activity by quantifying the blood GLT levels. An increase of switch activity along the disease course was clearly demonstrated in the peripheral group but not in the central group (Figure 6B; *p* = 0.013 vs. *p* = 0.48, *t*-test, more details in Section “Materials and Methods”). The activity boost was compatible with the higher *TGFβ1* levels for the peripheral group at dengue onset (Figure 6A).

## Discussion

In this study, we applied a unified selection scheme among several data sets to find signatures of antibody repertoires. We took advantage of PCA to focus on a smaller set of contributing CDR-H3 clones and used SVC plus LR models to detect signature clusters out of those contributing clones. This pipeline successfully reproduced the infection signature of chronic hepatitis B carrier children (Figure S1B in Data Sheet S1 in Supplementary Material) (13) and identified previously reported IgG signatures of dengue fever in South America (Figures 1 and 2) (11, 12). Although cluster ARQRGNWFDS was more prevalent in Mexico than in Nicaragua or Taiwan, cluster ARLDYYYYYGMDV was detectable in all three areas (Figures 1 and 2). It is not clear why cluster distributions differ with geographic locations. Potential biological factors could include ethnic groups, viral strains, or even transmission vectors. However, the possible technical biases from processing pipelines could not be ruled out, either. The intrinsic stochastic nature of human antibody responses to viral infections could also play an important role. More signatures from a wider coverage of dengue-endemic areas might provide a comprehensible pattern to address the question.

IgA has been proposed as a diagnostic approach to dengue fever (39, 40). Recently, the relationships between IgA and hemorrhages have also been reported in several studies (41–44). However, it is still not clear what mechanism causes the bleeding phenotype. In this study, we found seven IgA clusters that were closely associated with hemorrhages (Figure 5). Antigenic stimulations would likely account for the appearances of these CDR-H3 clusters. With recent advances in single-cell sequencing, it would be possible to get complete sequences of relevant monoclonal antibodies carrying these signatures, including the identities of light chains. Monoclonal antibodies accordingly reproduced would be of great use in addressing the underlying pathophysiology of dengue hemorrhages. A similar approach could be taken for other IgA-related bleeding disorders such as Henoch-Schönlein purpura or IgA nephropathy.

Innate IgA reactivity is an important contributing factor to shape IgA repertoires in mice (45). If the same is true for humans, the IgA specificity within human milk could also play a similar role. The corollary to the assumption would imply the compositions of IgA repertoires among dengue-infected infants would be affected by the prior IgA specificity within ingested milk. Thus, dengue-experienced mothers would influence their babies’ reactions to dengue viruses in an IgA-dependent manner. Existence of dengue-primed IgA in maternal milk could make these young kids prone to IgA-related bleeding disorders by differentially shaping their IgA repertoires. Such an effect would be absent from dengue-naive mothers. It would be intriguing to see if this hypothesis could address the unexplained dengue hemorrhages in young infants born to dengue-inflicted women. An easy prevention might be achievable by strict formula feeding for these young children.

In this study, we took healthy controls from another database to contrast immune repertoires from dengue patients. With this approach, we did reveal common infection signatures among dengue patients in both North or Central America and Taiwan (Figures 1 and 2). It would be useful if we could expand the collections of immune repertoires from even more clinical scenarios. With more sequences available, we would expect to discover relevant information from disease in other categories as well. The identified sequences might play essentials roles under those circumstances. The reusability of these repertoire data could *de facto* expand the possibility of sequence-based medicine to a new horizon.

In sum, we developed a unified pipeline to extract signatures of antibody repertoires and used dengue fever as a model disease to gain insights in infection- and phenotype-specific contexts. The results well exemplified the stochastic nature of reacting IgG repertoires and uncovered cluster-yielding characters of IgA repertoires in dengue hemorrhages. Differentials of class-switch activity and isotype effectors in humoral immunity could be new factors to consider in viral infections. For inflammatory diseases without animal models or known pathogens, the framework could be valuable in offering perspectives not derivable elsewhere.

## Ethics Statement

The protocol in recruiting dengue subjects was approved by the Institutional Review Board of Kaohsiung Medical University Hospital. Some of non-dengue controls were from a previous study, the protocol of which was approved by both the Institutional Review Board of National Health Research Institutes and the Institutional Review Board of En Chu Kong Hospital. Study methods were carried out in accordance with the relevant guidelines. Informed consents were obtained from all attendants.

## Author Contributions

C-HH, C-YL, H-CK, Y-JH, Y-WW, and J-YC helped in recruiting subjects. Y-HC and C-HY prepared sequencing libraries. W-HW did virus serotyping. S-FT, Y-HC, and H-HL designed the study. H-HL analyzed the data, wrote the manuscript and programmed the Python scripts.

## Conflict of Interest Statement

The authors declare that the research was conducted in the absence of any commercial or financial relationships that could be construed as a potential conflict of interest.
