# Supplementary material for: Shared IgG Infection Signatures vs. Hemorrhage-Restricted IgA Clusters in Human Dengue: A Phenotype of Differential Class-Switch via TGFβ1
Source: Front Immunol. 2017 Dec 4;8:1726. doi: 10.3389/fimmu.2017.01726 (PMC5723002; doi:10.3389/fimmu.2017.01726)
Supplement: Supplementary file 1 [file Data_Sheet_1.docx]

Supplementary Material

Shared IgG Infection Signatures *vs.* Hemorrhage-Restricted IgA Clusters in Human Dengue: a Phenotype of Differential Class-Switch *via TGFβ1*

Chung-Hao Huang^1,2†^, Ya-Hui Chang^6†^, Chun-Yu Lin^1,2^, Wen-Hung Wang^1^, Hui-Chung Kuan^7^, Ya-Ju Hsieh^4^, Yu-Wei Wang^4^, Chung-Hsiang Yang^6^, Jhen-Yan Chiu^5^, Shih-Feng Tsai^6^, Yen-Hsu Chen^1,2,3^, Hong-Hsing Liu^6,7*^

^1^Division of Infectious Diseases, Department of Internal Medicine, Kaohsiung Medical University Hospital, Kaohsiung 80756, Taiwan.

^2^School of Medicine, Graduate Institute of Medicine, Sepsis Research Center, Kaohsiung Medical University, Kaohsiung 80708, Taiwan.

^3^Department of Biological Science and Technology, College of Biological Science and Technology, National Chiao Tung University, HsinChu 30010, Taiwan.

^4^Sinying Hospital, Tainan 73042, Taiwan.

^5^Hsieh Te Kuei Pediatric Clinic, HsinChu 30072, Taiwan.

^6^Institute of Molecular and Genomic Medicine, National Health Research Institutes, Zhunan 35053, Taiwan.

^7^Pediatrics, En Chu Kong Hospital, Sanxia 23702, Taiwan.

^†^These authors contributed equally to this work.

*** Correspondence:** hhliu@nhri.org.tw

**
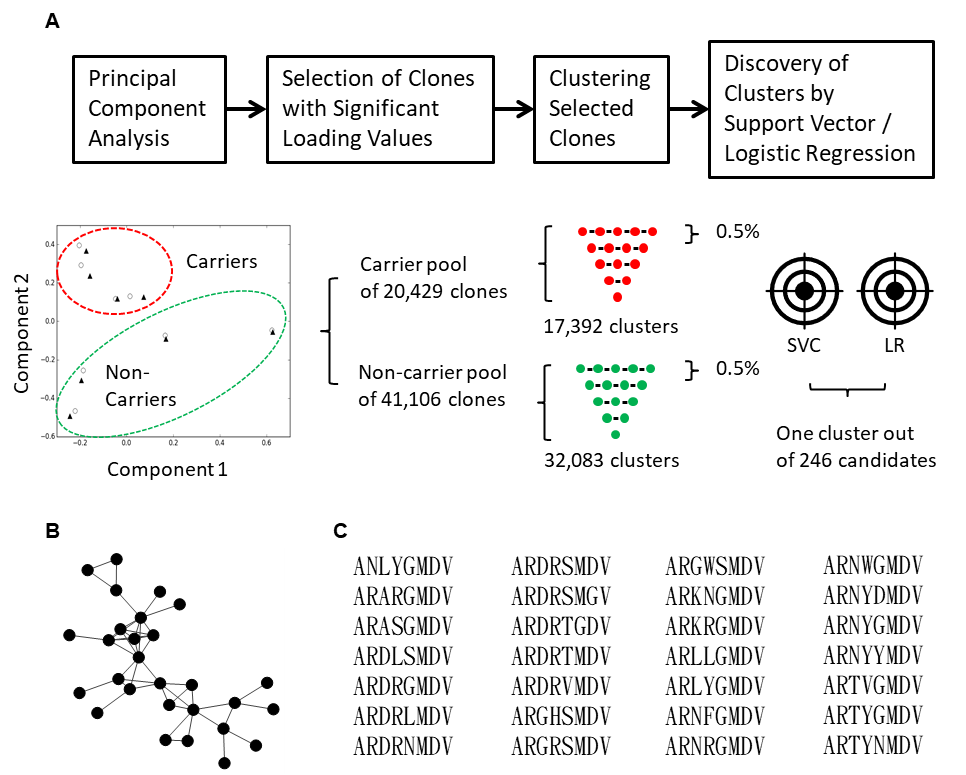
**

**Supplementary Figure 1 | Identification of a signature cluster upon chronic hepatitis B carrier children. (A)** Signature clusters were identified out of selected clones that bore significant loading values on PCA. Top 0.5% of clusters with most member counts were subjected to both SVC and LR selections, which yielded only one significantly supported cluster. **(B)** One 28-member cluster was discovered as the infection signature among carrier children of chronic hepatitis B. **(C)** The CDR-H3 sequences of the 28 members are listed.

**
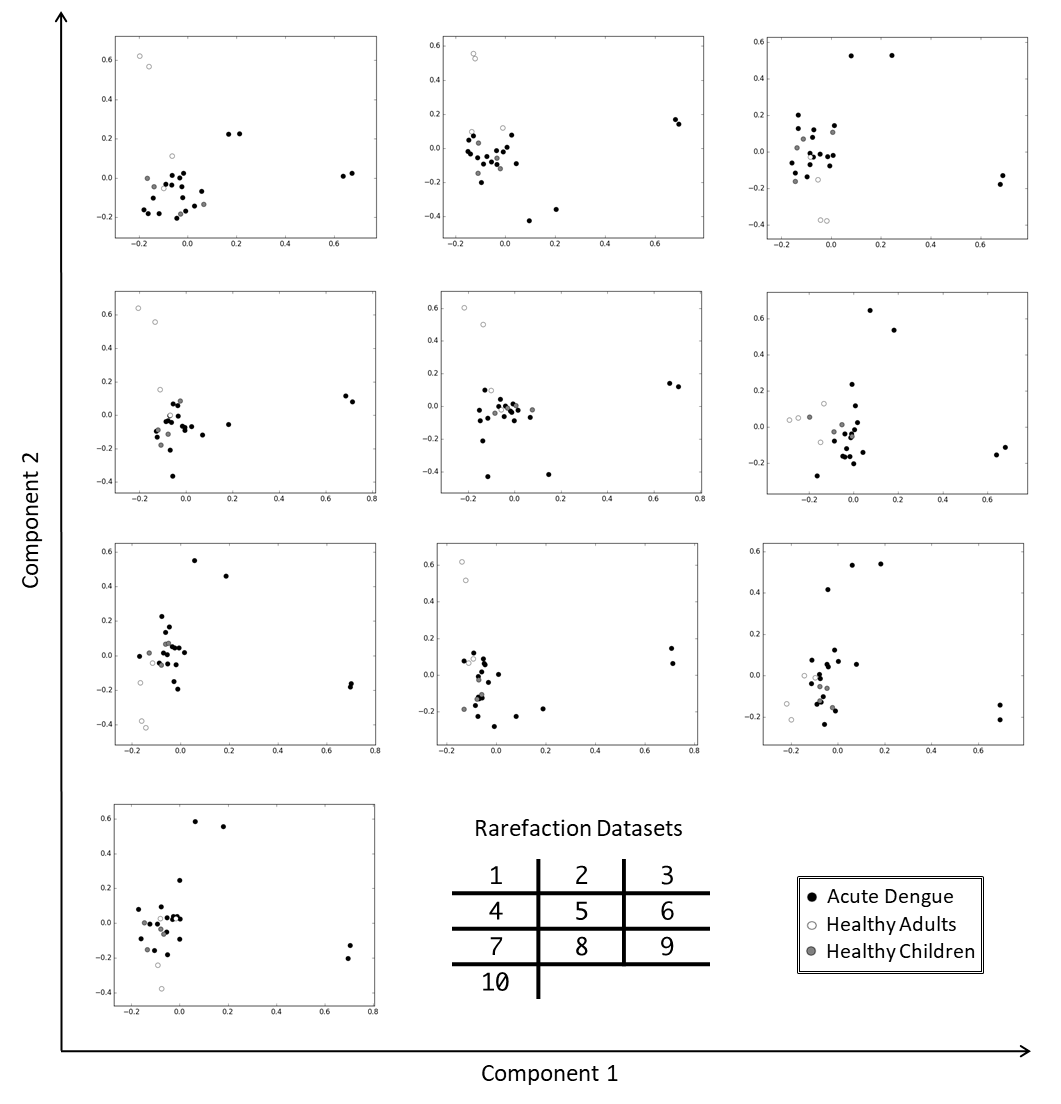
**

**Supplementary Figure 2 | PCAs of rarefied IgG immune repertoires.** 10 sets of randomly rarefied IgG immune repertoires comprising 19 acute Dengue samples from Mexico plus 4 healthy adults and 4 healthy children controls from Taiwan were subjected to principal component analyses. The acute Dengue group could be fairly separated from adult controls but not from children.

**
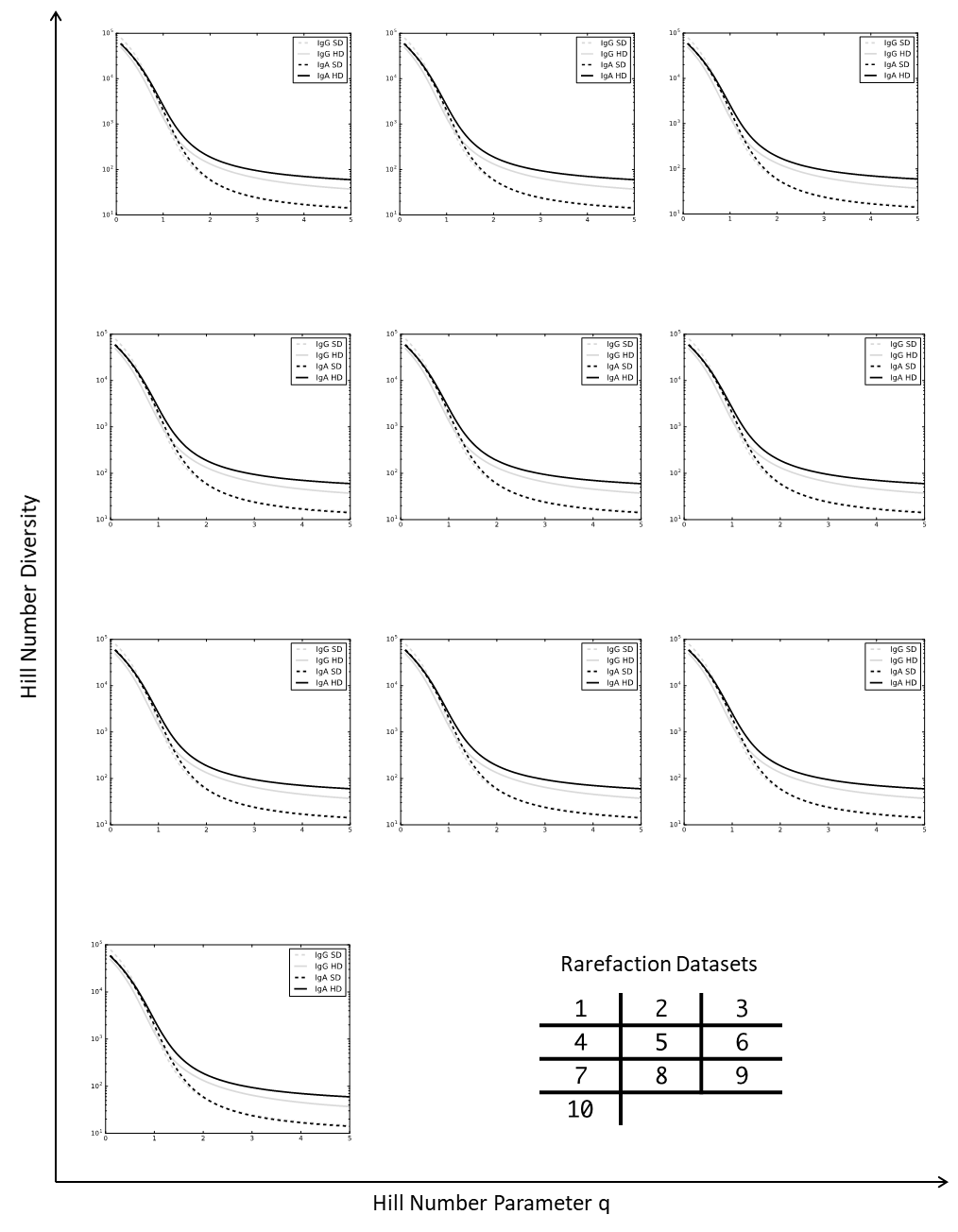
**

**Supplementary Figure 3 | Diversities of immune repertoires.** Profiles in Hill numbers were illustrated for IgG and IgA immune repertoires of each rarefied dataset. For both classes of immunoglobulins, hemorrhagic patients had higher diversities than non-hemorrhagic patients, as depicted by the upward trends of diversity curves. The differences were more prominent for IgA immune repertoires in all datasets.

**
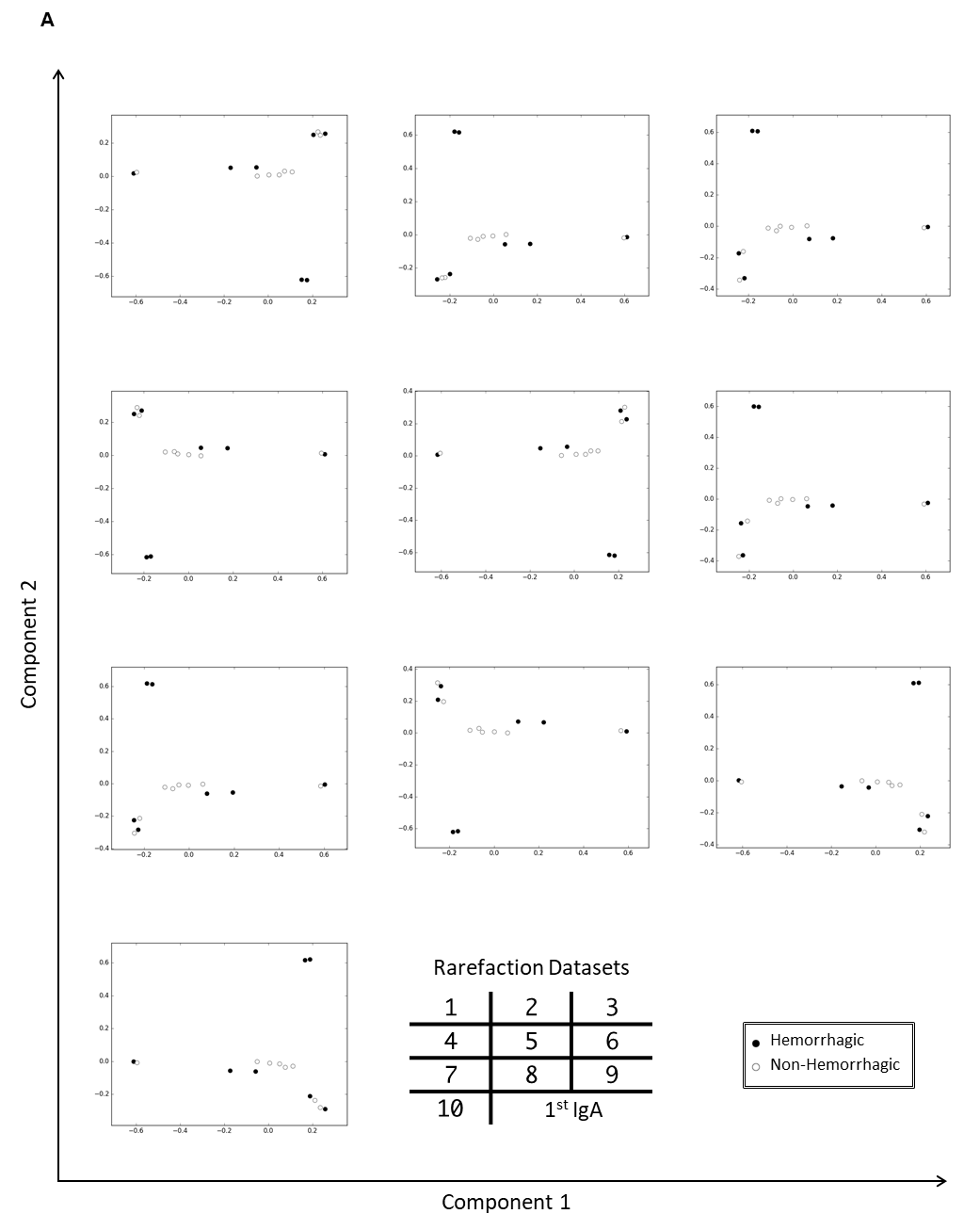
**

**Supplementary Figure 4 | PCAs of IgA immune repertoires. (A)** 1^st^ IgA immune repertoires from all rarefied datasets were subjected to PCA analyses. Separation of hemorrhagic patients from non-hemorrhagic patients was only suboptimal.

**
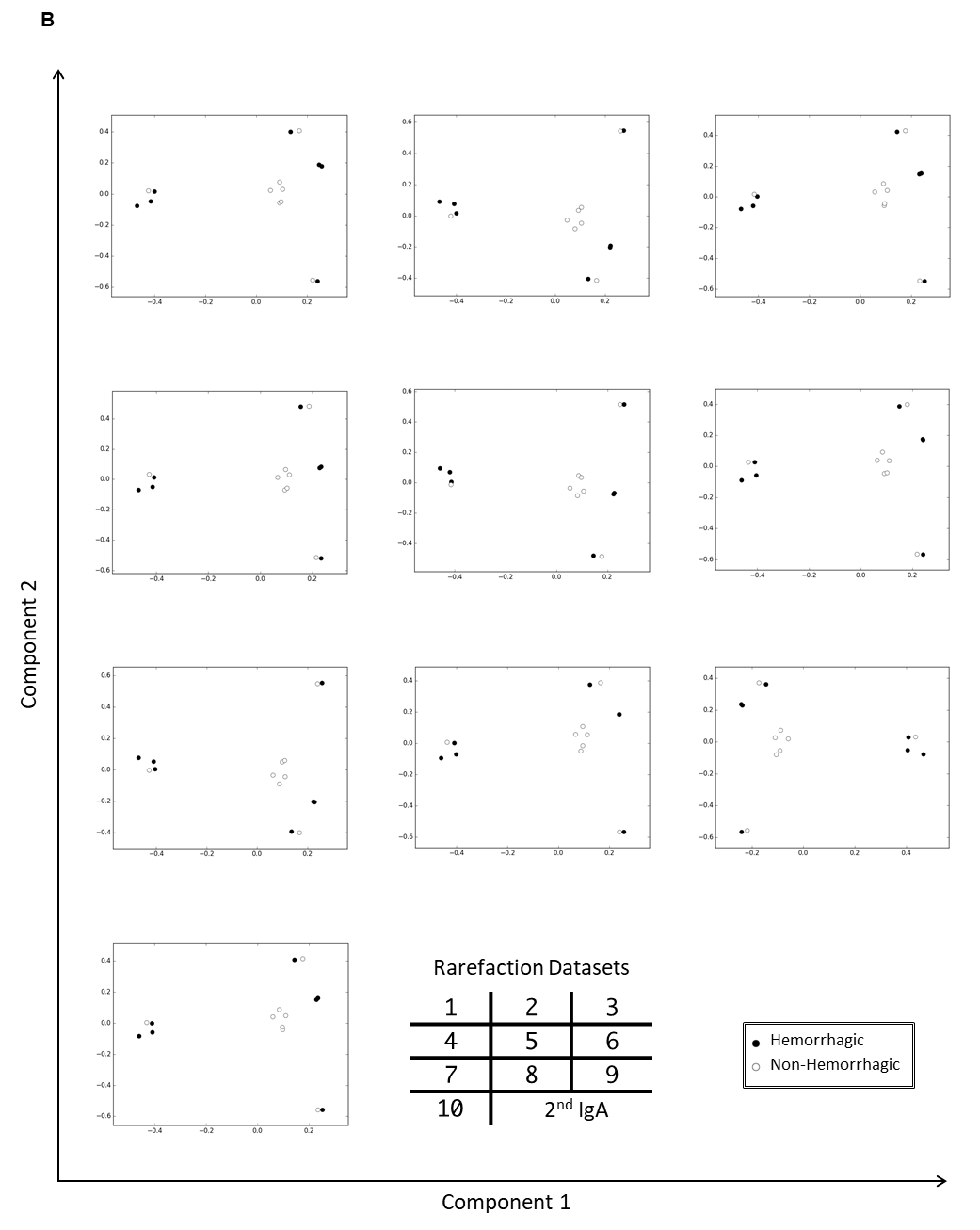
**

**Supplementary Figure 4 | Continued.** **(B)** PCA plots were prepared based on 2^nd^ IgA immune repertoires from all rarefied datasets. The clustering was not inferior to the result derived from complete repertoire sequences.
