# Supplementary material for: Shared IgG Infection Signatures vs. Hemorrhage-Restricted IgA Clusters in Human Dengue: A Phenotype of Differential Class-Switch via TGFβ1
Source: Front Immunol. 2017 Dec 4;8:1726. doi: 10.3389/fimmu.2017.01726 (PMC5723002; doi:10.3389/fimmu.2017.01726)
Supplement: Supplementary file 2 [file Data_Sheet_2.docx]

Supplementary Material

Shared IgG Infection Signatures *vs.* Hemorrhage-Restricted IgA Clusters in Human Dengue: a Phenotype of Differential Class-Switch *via TGFβ1*

Chung-Hao Huang^1,2†^, Ya-Hui Chang^6†^, Chun-Yu Lin^1,2^, Wen-Hung Wang^1^, Hui-Chung Kuan^7^, Ya-Ju Hsieh^4^, Yu-Wei Wang^4^, Chung-Hsiang Yang^6^, Jhen-Yan Chiu^5^, Shih-Feng Tsai^6^, Yen-Hsu Chen^1,2,3^, Hong-Hsing Liu^6,7*^

^1^Division of Infectious Diseases, Department of Internal Medicine, Kaohsiung Medical University Hospital, Kaohsiung 80756, Taiwan.

^2^School of Medicine, Graduate Institute of Medicine, Sepsis Research Center, Kaohsiung Medical University, Kaohsiung 80708, Taiwan.

^3^Department of Biological Science and Technology, College of Biological Science and Technology, National Chiao Tung University, HsinChu 30010, Taiwan.

^4^Sinying Hospital, Tainan 73042, Taiwan.

^5^Hsieh Te Kuei Pediatric Clinic, HsinChu 30072, Taiwan.

^6^Institute of Molecular and Genomic Medicine, National Health Research Institutes, Zhunan 35053, Taiwan.

^7^Pediatrics, En Chu Kong Hospital, Sanxia 23702, Taiwan.

^†^These authors contributed equally to this work.

*** Correspondence:** hhliu@nhri.org.tw

**
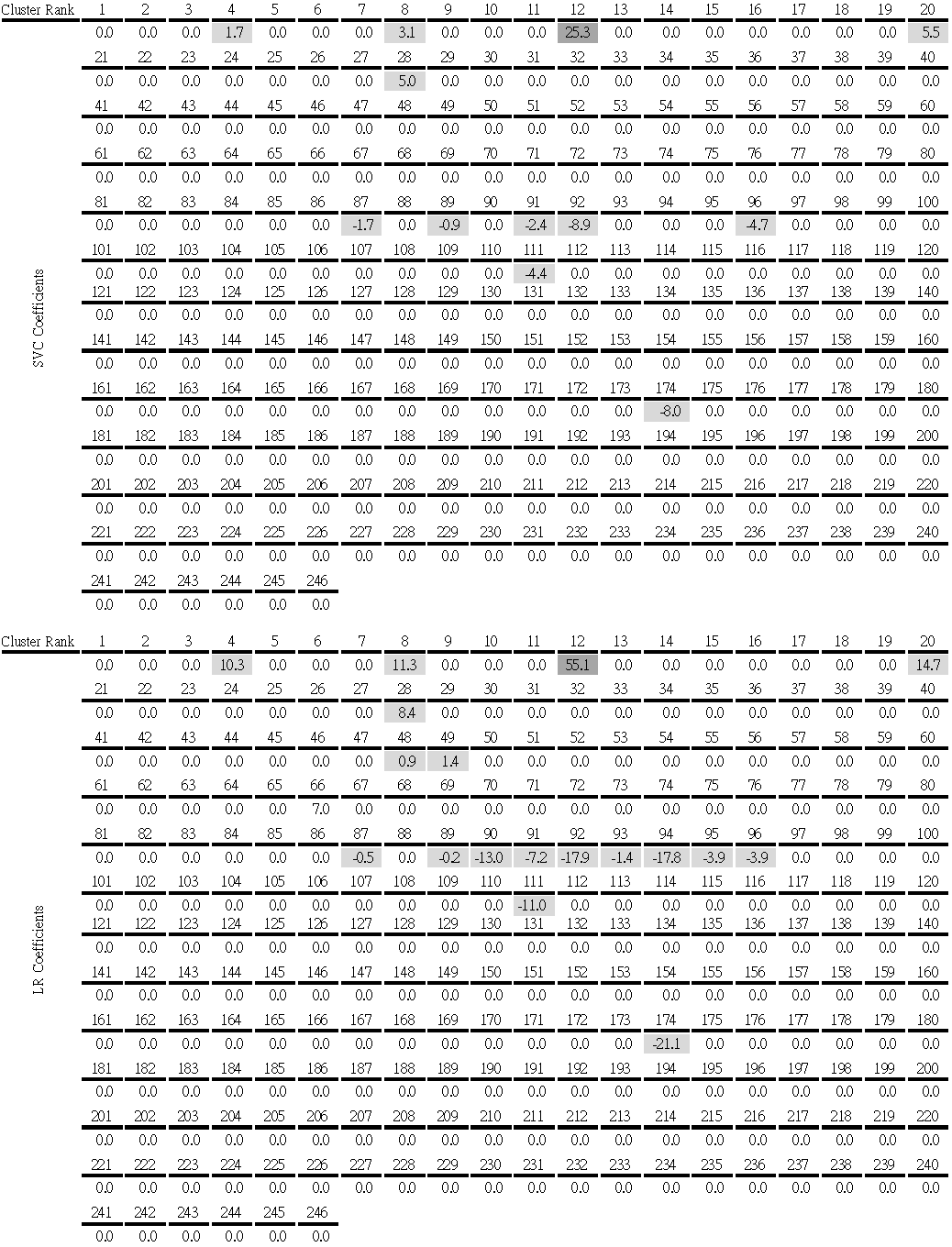
**

**Supplementary Table 1 | Coefficients of SVC and LR models in detecting infection signatures of IgG repertoires among chronic hepatitis B carrier children.** The top 0.5% of PCA-derived carrier and noncarrier clusters in terms of member counts were examined. Cluster 12, marked in dark grey, had significant positive coefficients in both SVC and LR models. Other less significant or negative coefficients in either model were colored in light grey. Both models agreed to each other very well.

**
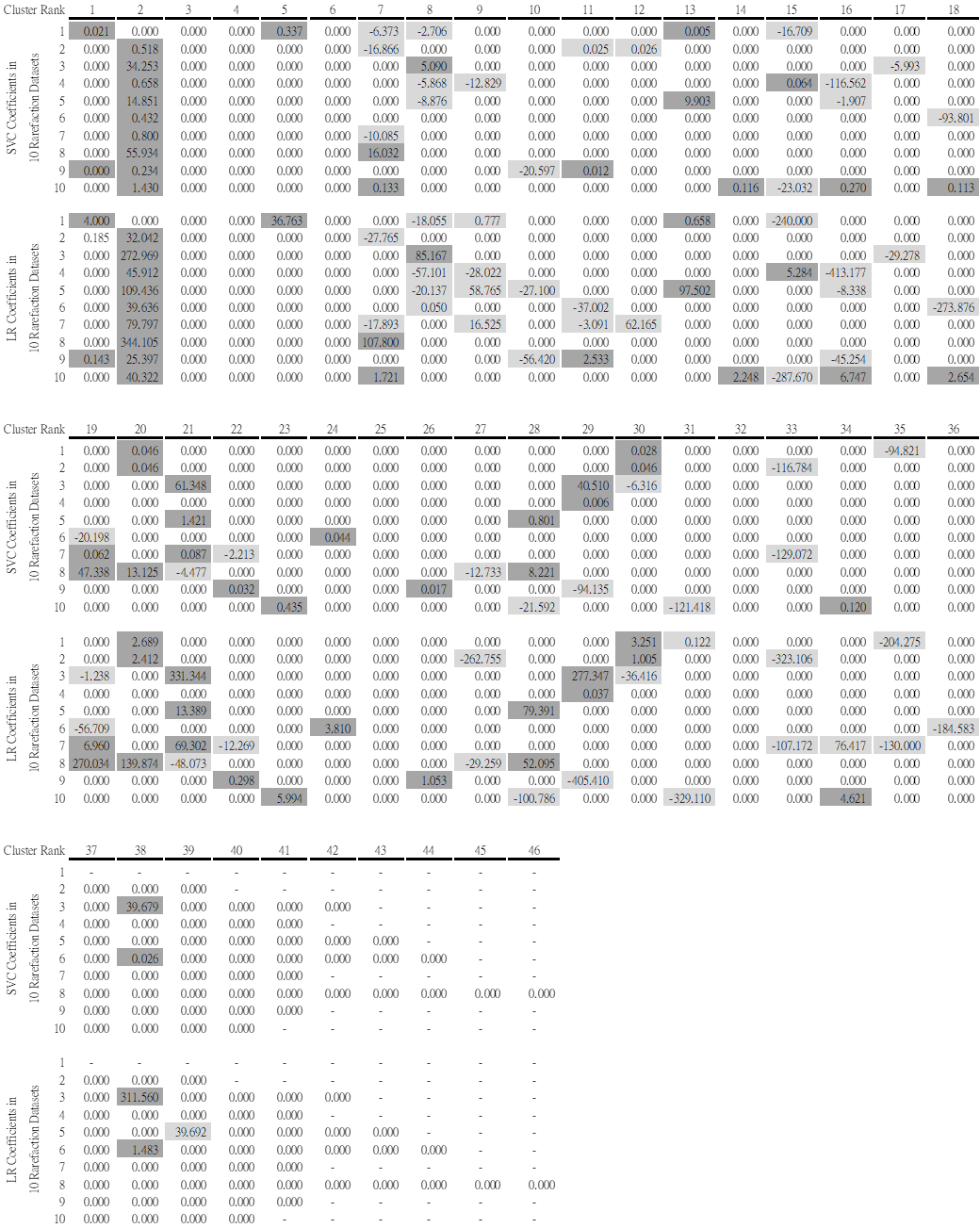
**

**Supplementary Table 2 | Coefficients of SVC and LR models in discovering signature clusters of IgG repertoires across all rarefied datasets in acute Dengue samples of Mexico.** The top 1% of PCA-derived clusters with higher member counts were tested. The mutually agreed clusters with positive coefficients by both models were shown in dark grey. Other discrepant or negative coefficients in either model were colored in light grey. Both models agreed to each other considerably well.

**
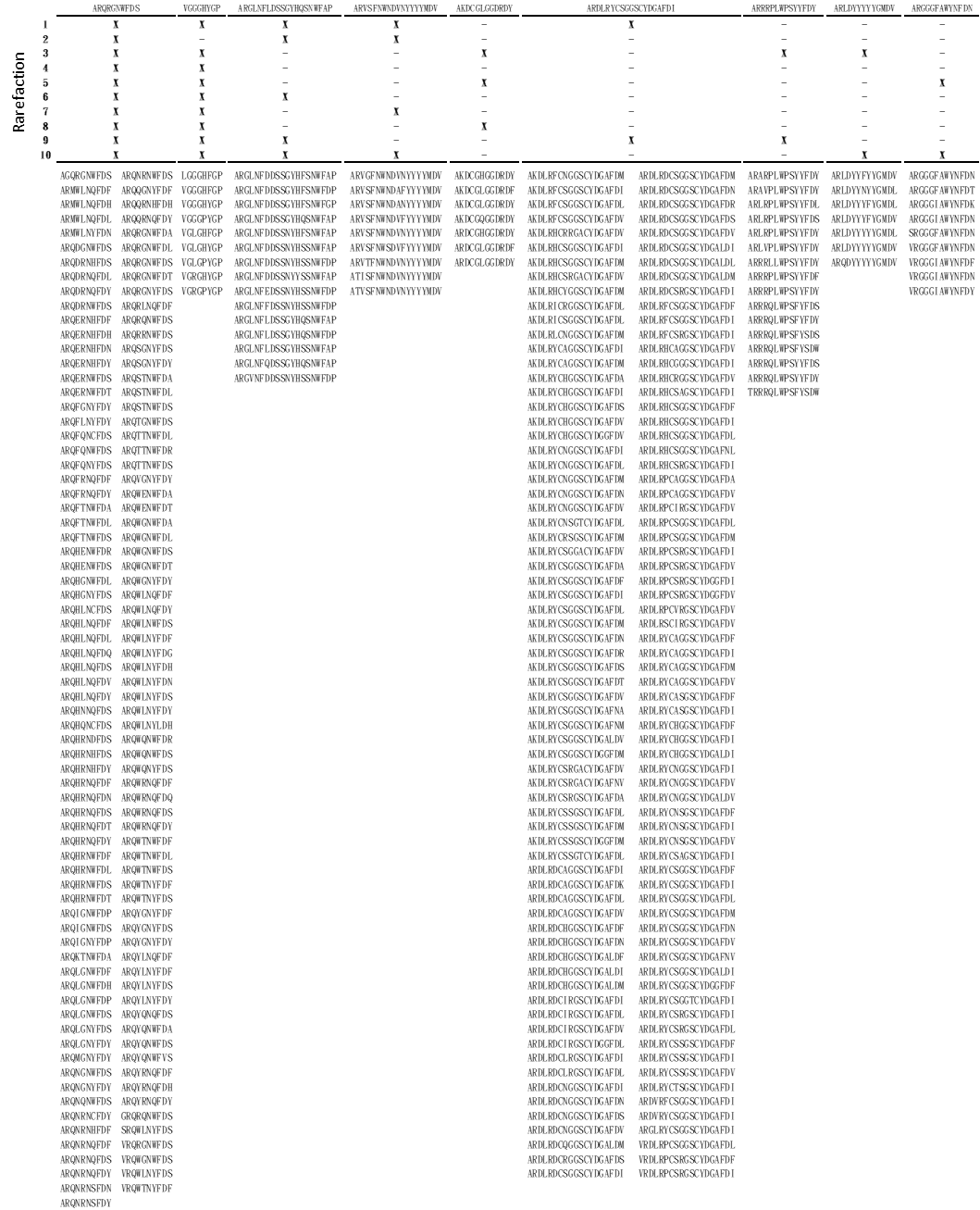
**

**Supplementary Table 3 | Clone sequences of the nine IgG clusters associated with acute Dengue fever in Mexico.** The representative sequences with the highest PageRank scores were listed above, and the complete sets were detailed at the bottom. ‘X’ marked presences in indicated rarefactions. Only those clusters with at least two counts were included.

**
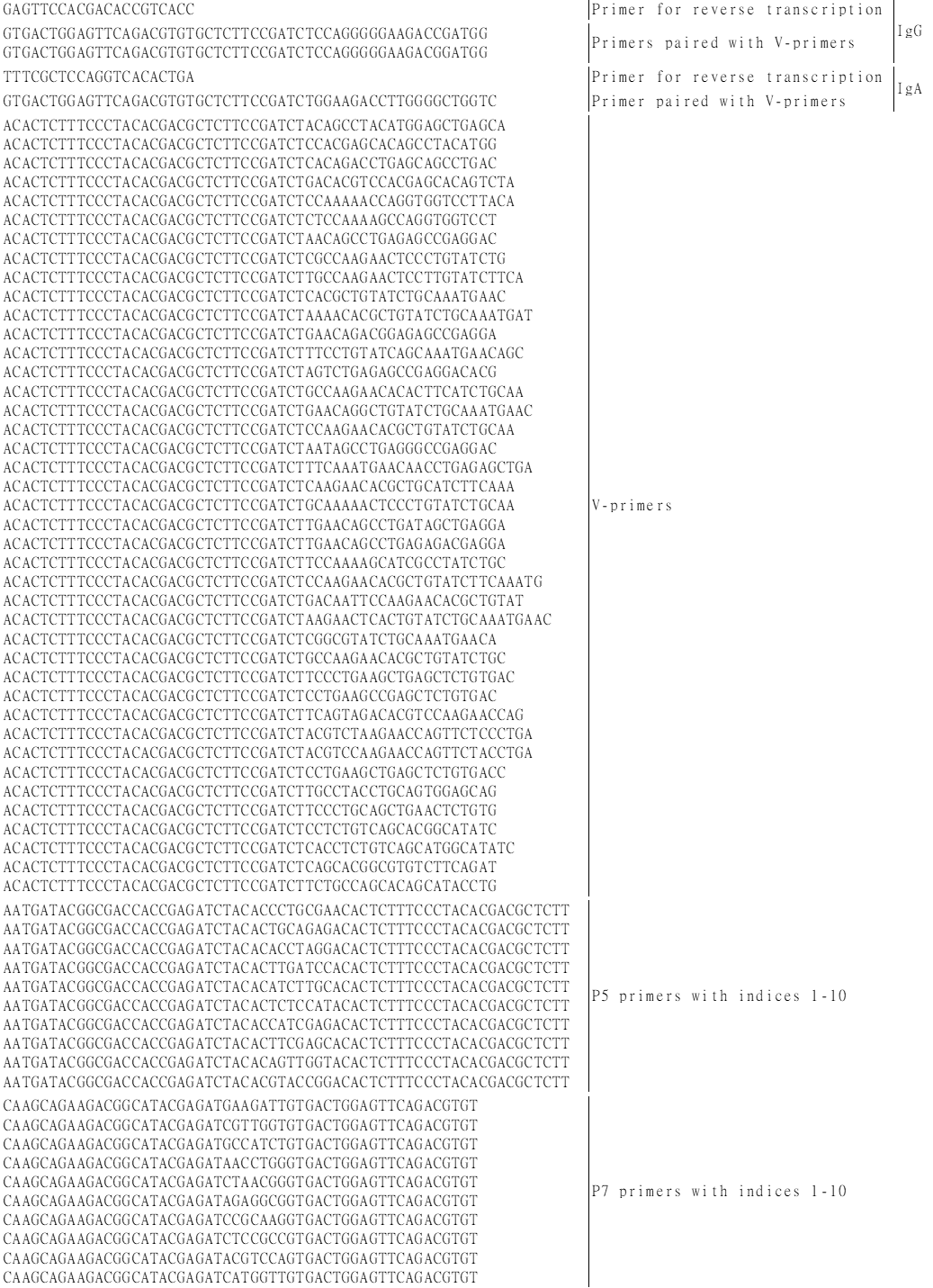
**

**Supplementary Table 4 | Oligonucleotides for preparation of NGS libraries.** Sequences of primers used in reverse transcription, repertoire preparation, and index incorporation were listed.

**Supplementary Table 5 | Sequence profiles of IgG and IgA immune repertoires.**

**
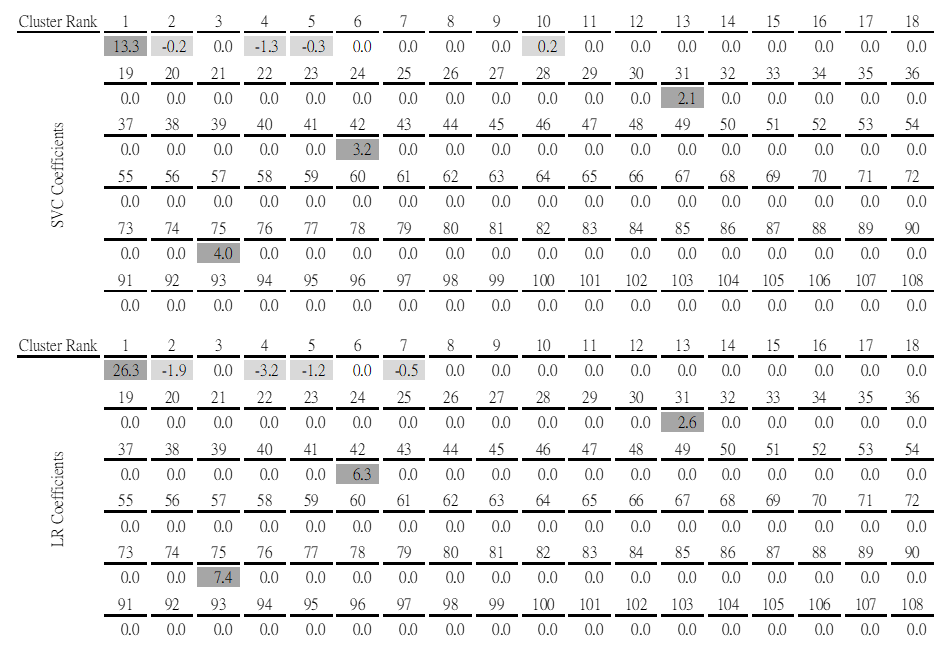
**

**Supplementary Table 6 | Coefficients of SVC and LR models in detecting infection signatures of IgG repertoires.** The top 1% of PCA-derived clusters with higher member counts were examined. The significant leading 4 clusters in SVC models were shown in dark grey and the corresponding LR coefficients were shown in the same hue. Other insignificant or negative coefficients in either model were colored in light grey. Both models agreed to each other very well.

**
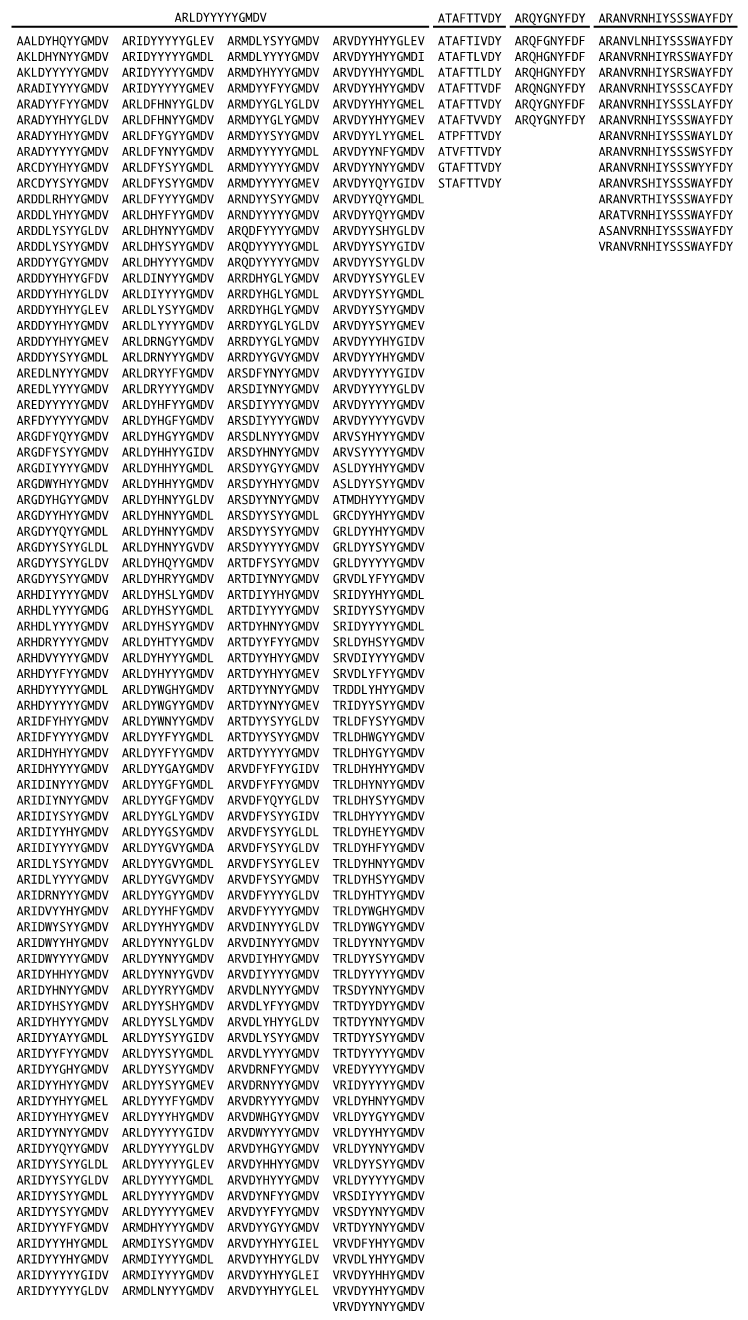
**

**Supplementary Table 7 | Clone sequences of the four signature clusters in IgG repertoires for Dengue infection.** The representative sequences with the highest PageRank scores were listed above. The complete sets were detailed at the bottom.

**
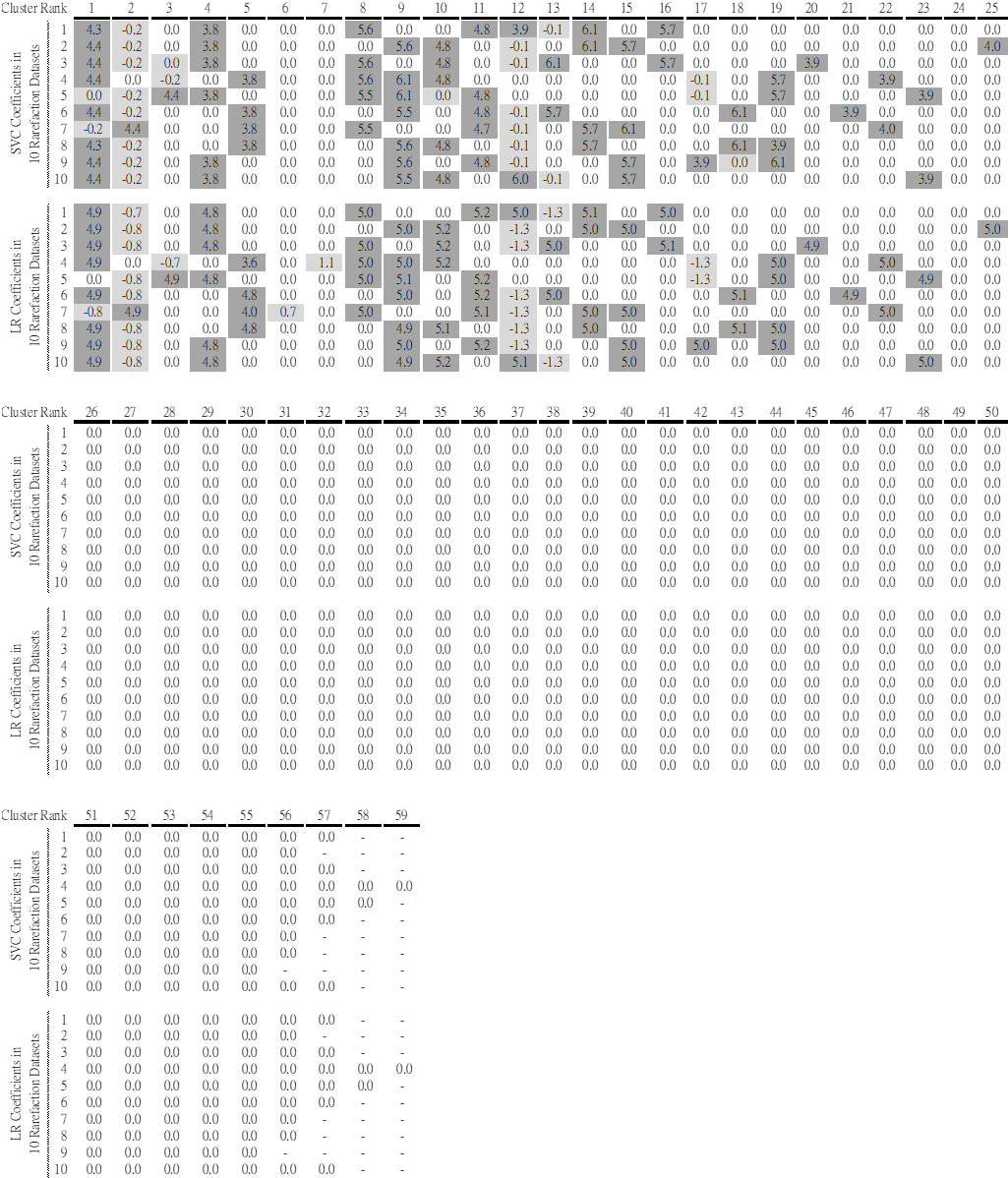
**

**Supplementary Table 8 | Coefficients of SVC and LR models in discovering hemorrhage-related clusters of IgA repertoires across all rarefied datasets.** The top 0.5% of PCA-derived clusters with higher member counts were tested. The significant leading 7 clusters in SVC models were shown in dark grey and the corresponding LR coefficients were shown in the same hue. Other insignificant or negative coefficients in either model were colored in light grey. Both models agreed to each other very well across all datasets.

**
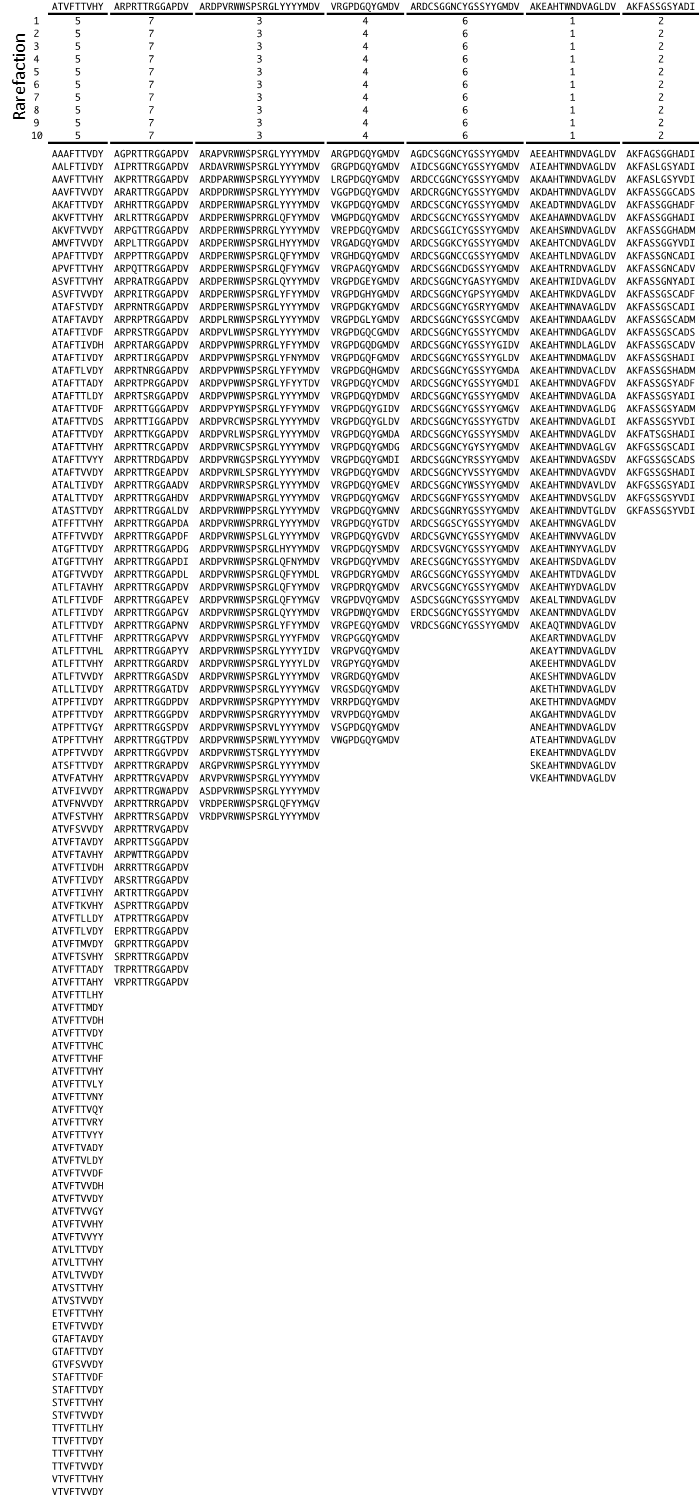
**

**Supplementary Table 9 | Clone sequences of the seven IgA clusters associated with hemorrhages in Dengue fever.** The representative sequences with the highest PageRank scores were listed above, and the complete sets were detailed at the bottom. The in between numbers denoted the ranks from high to low in the SVC models for each rarefaction.
